# Non-antibiotic compounds associated with humans and the environment can promote horizontal transfer of antimicrobial resistance genes

**DOI:** 10.1080/1040841X.2023.2233603

**Published:** 2023-07-18

**Authors:** Ilyas Alav, Michelle M. C. Buckner

**Affiliations:** Institute of Microbiology and Infection, College of Medical and Dental Sciences, University of Birmingham, Birmingham, UK

**Keywords:** Antimicrobial resistance, plasmid, horizontal gene transfer, conjugation, bacteria, pharmaceutical, pollutant, heavy metal

## Abstract

Horizontal gene transfer plays a key role in the global dissemination of antimicrobial resistance (AMR). AMR genes are often carried on self-transmissible plasmids, which are shared amongst bacteria primarily by conjugation. Antibiotic use has been a well-established driver of the emergence and spread of AMR. However, the impact of commonly used non-antibiotic compounds and environmental pollutants on AMR spread has been largely overlooked. Recent studies found common prescription and over-the-counter drugs, artificial sweeteners, food preservatives, and environmental pollutants, can increase the conjugative transfer of AMR plasmids. The potential mechanisms by which these compounds promote plasmid transmission include increased membrane permeability, upregulation of plasmid transfer genes, formation of reactive oxygen species, and SOS response gene induction. Many questions remain around the impact of most non-antibiotic compounds on AMR plasmid conjugation in clinical isolates and the long-term impact on AMR dissemination. By elucidating the role of routinely used pharmaceuticals, food additives, and pollutants in the dissemination of AMR, action can be taken to mitigate their impact by closely monitoring use and disposal. This review will discuss recent progress on understanding the influence of non-antibiotic compounds on plasmid transmission, the mechanisms by which they promote transfer, and the level of risk they pose.

## 1. Introduction

A key driver of antimicrobial resistance (AMR) is horizontal gene transfer, which allows for the exchange of genetic material, including antibiotic resistance genes, between bacteria (Dimitriu 2022; Darby et al. 2023). There are several different mechanisms of horizontal gene transfer in bacteria, including transduction, transformation and conjugation (Thomas and Nielsen 2005). Transduction involves the movement of genetic material mediated by phage. Transformation is the uptake of extracellular DNA by bacteria that can either be incorporated into the chromosome or co-exist as a plasmid (Johnston et al. 2014). The prerequisite for bacteria to undergo transformation is competence, which is regulated by genetic and environmental factors (Seitz and Blokesch 2013). In bacteria, transformation can play a role in the transfer of clinically relevant antibiotic resistance genes in various human pathogens (Winter et al. 2021). Genes encoding resistance to clinically relevant antibiotics are also commonly carried by plasmids, which are self-replicating pieces of DNA (San Millan 2018; Helinski 2022). These plasmids also often carry diverse functional gene groups, including partitioning systems, toxin–antitoxin systems, and conjugation machinery, to maintain plasmid stability and facilitate transmission amongst bacterial populations (Bouet and Funnell 2019; Virolle et al. 2020; Jurėnas et al. 2022). Conjugation is the primary, and most well-studied mechanism of plasmid transmission between cells and requires cell-to-cell contact mediated by conjugative pili or adhesins (Thomas and Nielsen 2005). Bacterial conjugation is a multi-step process initiated by expression of transfer genes, which encode components of the type 4 secretion system (T4SS), conjugative pili, and the relaxasome. The process of conjugation has been reviewed extensively elsewhere e.g. (Cabezon et al. 2015; Ilangovan et al. 2015; Berge et al. 2017; Koraimann 2018; Waksman 2019; Virolle et al. 2020; Shen et al. 2022), and is beyond the scope of this review.

Multi-drug resistant (MDR) bacteria often possess large conjugative plasmids carrying several antibiotic resistance genes conferring resistance to distinct classes of antibiotics (Rozwandowicz et al. 2018; Kopotsa et al. 2019). For example, the *bla*_NDM-1_ gene confers resistance to carbapenems (Kumarasamy et al. 2010). The *bla*_NDM-1_ gene and its variants are now disseminated globally across a range of Gram-negative bacteria on at least 20 different plasmid types (Johnson and Woodford 2013; Acman et al. 2022). As a last resort antibiotic, colistin is one of the few remaining effective treatment options for infections caused by carbapenem-resistant Enterobacteriaceae (Doi 2019). However, in 2015 the *mcr-1* gene encoding for colistin resistance was identified in the self-transmissible plasmid pHNSHP45 (Liu et al. 2016). Since then, ten variants of the *mcr* have been identified across virtually every continent on multiple plasmid backbones and host strains (Ling et al. 2020; Wang et al. 2020a). There are now reports of transmissible plasmids carrying both *bla*_NDM_ and *mcr* genes among other AMR genes like *tet*(X4), which confers resistance to tetracycline antibiotics, including the most recently approved tetracycline eravacycline (Zheng et al. 2016; Sun et al. 2019; Zhou et al. 2021; Lu et al. 2022b). Bacteria carrying such AMR plasmids pose a serious threat to animal and human health because of the limited treatment options (Camargo et al. 2015).

Some plasmids also carry genes encoding virulence determinants (Pilla and Tang 2018). For example, the large virulence plasmid pWR501 is crucial for *Shigella* spp. to cause dysentery and the pB171 virulence plasmid is involved in adhesion of enteropathogenic *Escherichia coli* (Sengupta and Austin 2011). In *K. pneumoniae*, MDR and hypervirulence are generally observed in distinct bacterial populations (Arcari and Carattoli 2022). However, multiple reports of strains carrying plasmids with MDR and hypervirulence determinants have been detected across the globe (Gu et al. 2018; Huang et al. 2018; Lam et al. 2019; Ahmed et al. 2021; Xie et al. 2021; Biedrzycka et al. 2022; Jia et al. 2022). Therefore, there is an urgent need to identify the drivers of plasmid transmission to reduce the spread and prevalence of antimicrobial resistance and virulence genes.

It is well established that sub-inhibitory concentrations of antibiotics in clinical and environmental settings provide selective pressure for bacteria to develop AMR (Gullberg et al. 2011; Chow et al. 2021; Ramsay et al. 2021; Sanchez-Cid et al. 2022). Antibiotic-mediated selection can also modulate conjugation dynamics by promoting or preventing plasmid transmission (Lopatkin et al. 2016). Multiple studies reported sub-inhibitory concentrations of antibiotics can promote conjugative plasmid transfer (Zhang et al. 2013; Lu et al. 2017; Moller et al. 2017; Shun-Mei et al. 2018; Xiao et al. 2022; Ding et al. 2022a), whereas older studies reported certain antibiotics, (e.g. ciprofloxacin and novobiocin), possess plasmid curing properties (McHugh and Swartz 1977; Hooper et al. 1984; Weisser and Wiedemann 1985; Michel-Briand et al. 1986).

Although most studies have investigated the impact of antibiotic use as a driver of AMR, the effect of non-antibiotic compounds on the spread of AMR genes has only recently attracted interest. There is a growing body of evidence suggesting non-antibiotic compounds, such as clinically approved drugs, food additives, and environmental pollutants can also promote AMR plasmid transmission (Liu et al. 2020). In this review, the evidence for the role of non-antibiotic compounds in promoting plasmid transmission and their reported mechanisms of action will be discussed. We have used the available data to assess the risks posed by different compounds for increasing plasmid transmission. Lastly, we aim to highlight unanswered questions regarding the neglected role that non-antibiotic compounds play in the dissemination of AMR plasmids.

## 2. Impact of clinically approved drugs on plasmid transmission

Commonly prescribed drugs, including antidepressants, analgesics, anticancer drugs, lipid-modifying agents, and β-blockers, have all been reported to promote the transfer of AMR plasmids (Table S1). Antidepressants are one of the most prescribed drugs, used for the treatment of clinical depression, anxiety disorders, and sometimes chronic pain (Joint Formulary Committee 2022). Antidepressants reported to promote plasmid transmission at clinically relevant concentrations include selective serotonin reuptake inhibitors (sertraline, fluoxetine, and escitalopram), the serotonin-norepinephrine reuptake inhibitor duloxetine and the norepinephrine-dopamine reuptake inhibitor bupropion (Table S1). Ding et al. found sertraline was the most effective at increasing the conjugative transfer of IncP plasmid RP4 and IncC plasmid pMS6198A. Exposure to 1 and 10 µg/mL sertraline increased pMS6198A transmission by four-fold and RP4 transmission by greater than six-fold, respectively (Ding et al. 2022b).

A different study by the same group found sertraline was the most potent at increasing plasmid transformation. Exposure to 0.01 µg/mL sertraline increased the transformation ratio of the non-conjugative pWH1266 plasmid into *Acinetobacter baylyi* ADP1 upon exogenous addition by greater than 1.5-fold compared to the untreated control (Lu et al. 2022a). However, pWH1266 is a cloning vector, not a clinically or environmentally derived plasmid, hence it is not representative of real world settings (Hunger et al. 1990). The atypical antidepressant agomelatine did not have any significant impact on plasmid conjugation/transformation (Lu et al. 2022a; Ding et al. 2022b).

Analgesic drugs, including non-steroidal anti-inflammatory drugs (ibuprofen, naproxen, and diclofenac) and paracetamol (acetaminophen), are widely used to treat fever and pain (Joint Formulary Committee 2022). Wang et al. showed that exposure to 50 µg/mL diclofenac or naproxen significantly increased the transformation ratio of pWH1266 into *A. baylyi* ADP1 by greater than two-fold compared to untreated control (Wang et al. 2020d). In a different study, the same group showed naproxen was the most potent analgesic drug at increasing conjugation. Exposure to 0.005 µg/mL naproxen increased the conjugative transfer of RP4 from *E. coli* to *P. putida* by four-fold and pMS6198A in *E. coli* by greater than two-fold (Wang et al. 2021). This is well below the reported peak plasma concentrations of naproxen (Davies and Anderson 1997). In a follow-up study, Wang et al. investigated uptake of a GFP-tagged RP4 plasmid from *P. putida* by microbes in an activated sludge community, following treatment with non-antibiotic pharmaceuticals (Wang et al. 2022). In the ibuprofen-dosed group, *Corynebacterium, Cutibacterium, Pseudomonas* and *Sphingomonas*, and in the naproxen-dosed group, *Legionella*, *Pseudomonas* and *Stenotrophomonas*, were found in higher abundance in the transconjugant pool and the corresponding recipient community compared to untreated controls (Wang et al. 2022). These genera include several pathogenic species, such as *C. diphtheriae, C. acnes, L. pneumophila, P. aeruginosa* and *S. maltophilia,* and Wang et al. speculated the ability of these genera to readily acquire AMR plasmids upon exposure to non-antibiotic pharmaceuticals could have wider implications. In *E. coli*, 50 µg/mL paracetamol significantly increased the conjugation of *mcr-1* and *tet*(X4) containing clinical AMR plasmids by greater than two- and 1.5-fold, respectively (Jia et al. 2021). However, this is significantly above the reported maximum plasma concentration of paracetamol (Brett et al. 2012).

Other clinically approved drugs that promoted plasmid conjugation or transformation included the lipid-lowering drug gemfibrozil, the β-blocker propranolol, the anti-epileptic drug carbamazepine, and the anti-cancer drug paclitaxel (Table S1). Wang et al. found that 0.05 µg/mL gemfibrozil and propranolol increased the transformation frequency of the non-conjugative cloning plasmid pWH1266 into *A. bayly*i by greater than two-fold (Wang et al. 2020d). A different study by the same group found 0.05 µg/mL gemfibrozil increased the conjugation frequency of RP4 from *E. coli* to *P. putida* by greater than seven-fold and pMS6198A between *E. coli* by greater than two-fold (Wang et al. 2021). These concentrations are well below the reported human peak plasma concentrations (Mohanalakshmi et al. 2021).

The anti-epileptic drug carbamazepine is also potent at increasing conjugative plasmid transfer. Exposure to 0.05–5 µg/mL carbamazepine increased the conjugative transfer of RP4 between *E. coli* and *P. putida* by greater than four-fold (Wang et al. 2019). The maximum plasma concentration of carbamazepine is between 4 to 12 µg/mL, (Nolen et al. 1988), meaning it may pose a risk for the spread of AMR genes in clinical settings. At 50 µg/mL, carbamazepine increased the conjugation frequency of GFP-tagged RP4 from mCherry-tagged *P. putida* to an activated sludge community by greater than seven-fold (Wang et al. 2022). However, this is significantly above carbamazepine concentrations in wastewater treatment plants (0.0037–0.0063 µg/mL) (Zhang et al. 2008), suggesting carbamazepine is unlikely to promote the spread of AMR genes in the environment. Recently, 0.1 µg/mL of the chemotherapeutic drug paclitaxel was found to increase the conjugative transfer of *tet*(X4)-carrying plasmids in *E. coli* by greater than two-fold (Yang et al. 2022). This is well below the peak plasma concentration of paclitaxel (Stage et al. 2018).

However, clinically approved drugs do not only increase plasmid transmission. For example, the anti-HIV drugs abacavir and azidothymidine reduced transmission of pCT_CTX-M_ among ST131 *E. coli* and pKpQIL among *K. pneumoniae* (Buckner et al. 2020). The antipsychotic drug chlorpromazine can also reduce plasmid transmission in *E. coli* (Mandi et al. 1975; Molnar et al. 1976; Buckner et al. 2020), and *K. pneumoniae* (Buckner et al. 2020). Therefore, the influence of non-antibiotic pharmaceuticals on plasmid transmission can vary depending on their physicochemical properties and potential biological effects within bacteria. The anti-plasmid properties of clinically approved drugs and other compounds has been reviewed elsewhere (Buckner et al. 2018; Getino and de la Cruz 2018), and will not be discussed in depth in this review.

## 3. Impact of food additives on plasmid transmission

As low-calorie or calorie-free alternatives to sugar, artificial sweeteners are used in numerous food and personal care products (Bandyopadhyay et al. 2008). However, artificial sweeteners have come under scrutiny for their potential biological footprint, such as metabolic and genotoxic effects (Bandyopadhyay et al. 2008). Some evidence suggests common food additives play a role in promoting plasmid transmission (Table S1). Yu et al. found that of the tested common non-nutritive sweeteners, sucralose was the most potent at enhancing the transformation frequency of several plasmids. At a concentration of 0.3 µg/mL, sucralose increased the transformation frequency of the non-conjugative pWH1266 plasmid into *A. baylyi* ADP1 by greater than two-fold (Yu et al. 2022). Sucralose also had a similar effect on the transformation frequency of pWH1266 into the Gram-positive bacterium *Bacillus subtilis* and the transformation frequency of GFP-tagged IncP-1 plasmid pKJK5 into mouse fecal bacteria (Yu et al. 2022). Similarly, 30 µg/mL acesulfame K increased the intra- and inter-genera conjugative transfer of RP4 by greater than three-fold (Yu et al. 2021).

Food preservatives are commonly used food additives and have been linked to enhancing conjugative plasmid transfer (Table S1). Mc Mahon et al. found food preservation methods, including low pH (pH ∼4), low (5 °C) or high (47 °C) temperatures, and high salt concentration (4% NaCl), significantly increased plasmid transfer rates in *E. coli* and *S. enterica* (Mc Mahon et al. 2007). For instance, the transfer rate of IncI1 plasmid TP307 in *E. coli* increased from 6.8 × 10^−12^ in LB broth to 1.2 × 10^−5^ in LB broth supplemented with 4% sodium chloride (Mc Mahon et al. 2007). Cen et al. found the food preservative sodium nitrite was the most potent at increasing the conjugative transfer of pCM184-Cm in *E. coli*, causing a greater than two-fold increase at 5 µg/mL concentration (Cen et al. 2020). However, pCM184-Cm is not a self-transmissible plasmid. In this system, the donor *E. coli* strain carrying pCM184-Cm possessed the transfer genes from the RP4 plasmid in its chromosome, enabling transfer of the pCM184-Cm plasmid.

## 4. Impact of environmental pollutants on plasmid transmission

Like antibiotics, pollutant residues from various sources are prevalent in environmental microbial communities, and potentially result in the emergence/transmission of AMR (Alderton et al. 2021). Common environmental pollutants include pharmaceuticals, personal care products, food, agricultural and industrial products, and plastics. Once these compounds enter the environment, they can be bio-accumulative, persistent, and constantly replenished due to anthropogenic activities (Cousins et al. 2019; Gomes et al. 2020; Alderton et al. 2021). There is now evidence from multiple studies suggesting a relationship between some common environmental pollutants and horizontal transfer of AMR genes (Table S2).

Glyphosate is one of the most extensively used broad-spectrum herbicides and crop-desiccants for agricultural weed control and has the potential to leach from agricultural soils into drainage water and wastewater (Gillezeau et al. 2019). 0.6 µg/mL glyphosate increased the transmission of an unnamed AMR plasmid in *E. coli* by more than four-fold without affecting cell growth or viability (Zhang et al. 2021). This is within the range of reported glyphosate concentrations of 0.43 µg/mL and 0.7 µg/mL in surface waters of the US and Argentina, respectively (Peruzzo et al. 2008; Van Bruggen et al. 2018).

Bisphenols are synthetic chemicals used to manufacture plastics, such as polycarbonates and epoxy ester resins (Liu et al. 2021). Plastic waste and industrial waste from plastic manufacturing releases bisphenols into the environment including soils, rivers, and marine environments (Liu et al. 2021). Recently, Feng et al. reported that 10 and 100 µg/L bisphenol S increased the conjugative transfer of RP4 by greater than four-fold between *E. coli* and from *E. coli* to *S. enterica*, respectively (Feng et al. 2022). These concentrations are within the reported bisphenol concentrations in sediment, surface water, and sludge (Qiu et al. 2019) and detected levels in bodily fluids from occupational exposure (Ribeiro et al. 2017). Despite their widespread presence in the environment, the impact of many other herbicides and plastic pre-cursors on AMR gene transfer remains unknown.

Disinfection by-products are environmental pollutants released into rivers and lakes from wastewater treatment plants (Krasner 2009). He et al. found that exposure to 10 µg/mL dichloroacetonitrile increased conjugation of RP4 in *E. coli* by more than five-fold (He et al. 2022). Nitric oxide (NO) is another by-product of wastewater treatment processes (Law et al. 2012) and is released into rivers, lakes, sediments, and soil during the nitrogen cycle (Fowler et al. 2013). Huang et al. used sodium nitroprusside, an NO donor, to simulate the process of sustained NO release during water treatment. A 0.1 mM concentration of sodium nitroprusside (0.65 µM of released NO) increased RP4 conjugation by more than nine-fold between *E. coli* and from *E. coli* to *S. enterica* (Huang et al. 2022). Dyeing wastewater from the cosmetic, food, pharmaceutical, and textile industries is another source of environmental pollution (Al-Tohamy et al. 2022). Jiao et al. found that the conjugative transfer of RP4 in *E. coli* was the highest when exposed to 0.8 µg/L *o*-xylene, which was 219-fold higher than that of the untreated control (Jiao et al. 2017). Typically, dyeing wastewater is treated before release into waterways. However, dyeing by-products, such as *o*-xylene, can still leach into the environment and have been detected at concentrations of up to 6.8 µg/L in surface waters (Duan et al. 2017; Yaseen and Scholz 2019).

Engineered nanomaterials, such as nanoalumina, have a diverse range of applications including drug delivery, materials manufacturing, cosmetic fillers, and catalysis, owing to their unique electronic, mechanical, and thermal properties (Baig et al. 2021). Qiu et al. reported that exposure to 5 mM nanoalumina increased the conjugative transfer of RP4 between *E. coli* by greater than 100-fold compared to untreated control (Qiu et al. 2012). Like nanomaterials, ionic liquids are another group of compounds with emerging applications in chemistry, chemical engineering, environmental science, materials science, and medicine (Kaur et al. 2022). Several ionic liquids can enhance plasmid transmission (Table S2). At 1 mg/mL concentration, the ionic liquid BMIM-PF_6_ was effective at increasing the conjugative plasmid transfer of RP4 and the IncP plasmid RK2 by 60-fold compared to untreated control (Wang et al. 2015; 2020c). Additionally, ionic liquids enriched for transconjugants including pathogenic species like *A. baumannii* and *S. enterica* (Wang et al. 2020c). Recently, a study reported that bacteria exposed to 10 µg/mL carbon nanotubes showed greater than a five-fold increase in the conjugative transfer of IncP-1 plasmid pB10 (Table S2) (Weise et al. 2022). Several studies demonstrate marine microplastics provide a platform for microbial colonization, biofilm formation, and horizontal gene transfer (Arias-Andres et al. 2018). For instance, the conjugation frequency of GFP-tagged pJKJ5 from *E. coli* to *Pseudomonas* sp. increased from 2.5 ± 2.9 × 10^−6^ in untreated water to 8.2 ± 9.0 × 10^−3^ in microplastic treated water (Table S2) (Arias-Andres et al. 2018). Biofilm formation has been suggested to promote plasmid transmission owing to the proximity of cells to one another (Hennequin et al. 2012; Stalder and Top 2016; Element et al. 2023), therefore, biofilms on microplastics could promote horizontal gene transfer.

## 5. Impact of heavy metals on plasmid transmission

Heavy metals occur naturally in the environment at very low concentrations. However, sources of heavy metal pollution, such as mining, petrochemical plants, pesticide production, and untreated sewage, can cause environmental accumulation (Jin et al. 2020). Heavy metal resistance determinants are present in virtually all bacterial species and potentially provide cross-resistance to clinically relevant antimicrobials (Pal et al. 2017). Mobile genetic elements, such as transposon Tn*21*, can carry heavy metal resistance genes alongside AMR genes, which can be mobilized onto plasmids (Liebert et al. 1999). These can accumulate and become widely disseminated in Gram-negative populations through horizontal gene transfer (Partridge et al. 2018; Yang et al. 2018).

There are several lines of evidence for the potential role of various heavy metals in facilitating plasmid transmission (Table S2). Mercury has been consistently reported in several studies to enhance the conjugative transfer of different plasmids (Table S2). One study found that 1.36 µg/mL mercury chloride increased the conjugative transfer of GFP-tagged pKJK5 from *E. coli* to a sludge bacterial community by more than two-fold (Lin et al. 2019). Similarly, 0.1 µg/mL mercury chloride enhanced the conjugative transfer of RP4 in *E. coli* by more than five-fold (Li et al. 2022). At 100 µg/mL, the iron minerals hematite and ferrihydrite increased conjugative transfer of ColE1 replicon pRK2013 plasmid in *E. coli* by more than three- and four-fold, respectively (Table S2). On the other hand, at higher concentrations (≥1 mg/mL) iron minerals significantly reduced conjugative transfer of pRK2013 (Tang et al. 2022). However, the effect of the iron minerals on bacterial growth was not tested, therefore the reduction in conjugation frequencies at higher concentrations could also be due to growth inhibition. Recently, a study showed that 5 µg/mL arsenic increased the conjugative transfer of several carbapenemase/extended spectrum β-lactamase encoding AMR plasmids by at least four-fold (Table S2) (Kothari et al. 2023).

However, the effect some heavy metals have on plasmid transmission is not clear-cut. For example, cadmium and copper have been reported to increase the conjugation frequency of different plasmids (Table S2) (Zhang et al. 2018; 2019; Wang et al. 2020b; Pu et al. 2021). Whereas other studies reported exposure to cadmium and copper at similar concentrations resulted in a significant reduction in conjugative plasmid transfer (Suzuki et al. 2012; Lin et al. 2019; Buberg et al. 2020). Therefore, the effect of some heavy metals on plasmid transmission may depend on the bacterial species and plasmid type.

## 6. How do non-antibiotic compounds increase plasmid transmission?

**Figure 1.**
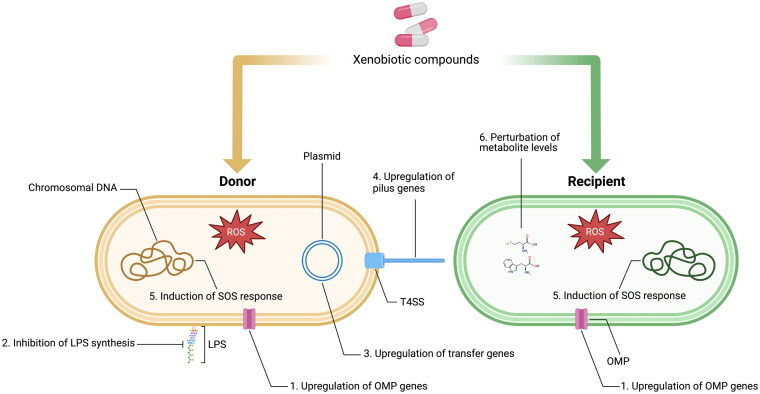
Reported mechanisms by which non-antibiotic compounds promote the conjugative transfer of plasmids in Gram-negative bacteria. Increased membrane permeability can be mediated by ***1)*** upregulation of genes encoding outer membrane proteins, or ***2)*** inhibition of LPS synthesis. ***3)*** Upregulation of transfer genes on conjugative plasmids, including plasmid transfer and replication, type 4 secretion system, and ***4)*** pilin-formation genes, can promote mating pair formation and stabilization to increase plasmid transfer. ***5)*** Overproduction of reactive oxygen species (ROS) leads to oxidative stress and the upregulation of the SOS response genes. This may enhance plasmid transmission by facilitating increased plasmid entry. ***6)*** Perturbed levels of metabolites, such as amino acids, have also been linked to enhanced plasmid transmission by affecting the expression of transfer genes. LPS, lipopolysaccharides; OMP, outer membrane protein; T4SS, type 4 secretion system. Created with BioRender.com.

Targeting plasmid transmission using anti-plasmid compounds is a potential strategy to reduce the dissemination and prevalence of AMR genes (Buckner et al. 2018; Getino and de la Cruz 2018). Therefore, it is important to understand the mechanism of how certain compounds alter plasmid transmission to allow potential targets for inhibition to be identified. Studies have proposed various mechanisms of how non-antibiotic compounds may enhance plasmid transmission in Gram-negative bacteria (Figure 1). These compounds affect various aspects of bacterial physiology and remove barriers to conjugation to increase plasmid transmission/transformation. There is currently a lack of data on the mechanistic details of such compounds in Gram-positive bacteria. Hence, the mechanisms discussed herein focus on Gram-negative bacteria.

### 6.1. Increased cell membrane permeability

One of the most reported mechanisms of increased plasmid transmission by non-antibiotic compounds is increased membrane permeability accompanied by cell membrane damage (Qiu et al. 2012; Zhang et al. 2018; Wang et al. 2019; Cen et al. 2020; Wang et al. 2020d; 2021; Yu et al. 2021; Cui et al. 2022; He et al. 2022; Yang et al. 2022; Lu et al. 2022a; Ding et al. 2022b). The cell membrane presents a barrier for conjugation, which requires close contact between the donor and recipient cells to allow for plasmid transmission (Samuels et al. 2000; Thomas and Nielsen 2005). Transient increase in membrane permeability induced by non-antibiotic compounds could be conducive to plasmid transfer between different bacteria. However, the impact and role of changes in membrane permeability on conjugative plasmid transfer has not been fully established.

### 6.2. Reduced cell-to-cell distance

Another effect by non-antibiotic compounds is reduced cell-to-cell distance. Using transmission electron microscopy, Qiu et al. found cells exposed to nanoalumina had altered morphology; cells were arranged more densely and were drawn closer to each other (Qiu et al. 2012). Wang et al. also reported similar findings when cells were exposed to diclofenac, gemfibrozil, ibuprofen, naproxen, and propranolol (Wang et al. 2021). Specifically, the average distance between adjacent cells decreased from 0.49 μm in untreated cells to 0.23 μm when exposed to these drugs (Wang et al. 2021). Multiple studies reported reduced distance and increased contact between donor and recipient cells when treated with disinfection by-products (dichloroacetonitrile and trichloromethane), sodium nitroprusside (NO donor), food preservatives (sodium nitrite, sodium benzoate, and triclocarban), and the anti-cancer drug paclitaxel (Cen et al. 2020; He et al. 2022; Huang et al. 2022; Yang et al. 2022). Since conjugation is a contact-dependent process (Thomas and Nielsen 2005), reduced cell-to-cell distance presumably increases the chances of a donor and recipient cell coming into contact for plasmid transfer. This is supported by evidence that higher conjugation frequencies are associated with biofilms, where cells are more densely distributed, compared to planktonic cells (Stalder and Top 2016; Element et al. 2023). However, the link between cell-to-cell distance and conjugation frequency has not been directly studied.

### 6.3. Expression of outer membrane proteins

Studies have also described increased expression of outer membrane protein (OMP) genes with exposure to compounds that promote conjugation, which is indicative of cell membrane remodeling (Rosas and Lithgow 2022). In donor bacteria, carbamazepine significantly upregulated expression of multiple OMP genes, including *ompA* and *ompN*, and increased abundance of OMPs, such as OmpF and OmpC (Wang et al. 2019). Similarly, food preservatives (sodium nitrite, sodium benzoate, and triclocarban), the anti-cancer drug paclitaxel, heavy metals (copper, chromium, silver, and zinc), the herbicide glyphosate, and disinfection by-products (dichloroacetonitrile and trichloromethane) significantly upregulated the expression of several OMP genes, such as *ompA*, *ompC* and *ompF*, in both donor and recipient bacteria (Zhang et al. 2018; Cen et al. 2020; Wang et al. 2020b; Zhang et al. 2021; He et al. 2022; Yang et al. 2022). Proteomic analysis of mating systems treated with diclofenac, gemfibrozil, ibuprofen, naproxen, or propranolol revealed the abundance of OmpC and OmpF proteins in donor *E. coli* cells, and OmpA, OprH, OprL and OprQ proteins in recipient *P. putida* cells were significantly increased (Wang et al. 2021). RNA-seq analysis confirmed the corresponding OMP genes were significantly upregulated compared to untreated control (Wang et al. 2021). Iopromide, which did not affect conjugative plasmid transfer, had no significant effect on cell membrane permeability or expression of OMP genes (Wang et al. 2021). Ding et al. found antidepressants increased expression of porin proteins (LamB, OmpC, and OmpF) and the outer membrane channel TolC in donor *E. coli* cells (Ding et al. 2022b). In the recipient *P. putida* cells, the abundance of OMPs, such as OprD, OprE, OpdC, and FecA, were also significantly higher (Ding et al. 2022b).

One possible mechanism by which increased abundance of OMPs could enhance plasmid transmission is through mating-pair formation. Low et al. demonstrated that mating-pair formation is stabilized by interactions between variants of the plasmid-borne OMP TraN of the donor cell and outer membrane receptors on the recipient cells (Low et al. 2022). For example, the TraNβ protein encoded by IncFII plasmid pKpQIL in the donor interacted with the porin OmpK36 (OmpC homolog in *E. coli*) in the recipient (Low et al. 2022). Therefore, the upregulation of OMP genes and increased abundance of OMPs may also enhance plasmid transmission by stabilizing mating pair formation between donor and recipient cells.

### 6.4. Reduced lipopolysaccharide synthesis

Lipopolysaccharides (LPS) on the outer membrane of Gram-negative bacteria also present a permeability barrier for conjugation due to steric hinderance (Bertani and Ruiz 2018). Early studies found the structure of LPS on the recipient’s surface is important for bacterial conjugation (Hoekstra and Havekes 1979; Sanderson et al. 1981; Duke and Guiney 1983). Huang et al. found that with exposure to the nitric oxide (NO) donor sodium nitroprusside, the membrane permeability of donor and recipient *E. coli* was significantly increased, but there was no change in the expression of OMP genes (Huang et al. 2022). Instead, the amount of LPS in both donor and recipient cells was reduced to 21-25% of untreated cells. This was correlated with significantly lower expression of LPS biosynthesis genes, such as *waaG, waaH*, and *waaJ* (Huang et al. 2022). This suggests increased permeability induced by NO was due to LPS deficiency.

### 6.5. Increased expression of transfer genes

Self-transmissible plasmids, including many AMR plasmids, carry all the genes encoding proteins required for the conjugation process (Norman et al. 2009; Smillie et al. 2010; Virolle et al. 2020). These genes are involved in the coordinated processes of plasmid replication, partitioning and conjugation (Virolle et al. 2020). Therefore, the rate of conjugation is regulated by the expression of transfer genes (Smillie et al. 2010). Multiple studies found that non-antibiotic compounds that increase plasmid transmission also upregulate the expression of various transfer genes in donor bacteria. Qiu et al. reported that increasing concentrations of nanoalumina significantly repressed the expression of major global regulatory genes *trbA* and *korA* in RP4, resulting in upregulation of the mating pair formation gene *trbBp* and the DNA transfer and replication gene *trfAp* (Qiu et al. 2012). Similarly, food preservatives (sodium nitrite, sodium benzoate, and triclocarban), disinfection by-products (dichloroacetonitrile and trichloromethane), and several clinically approved drugs (diclofenac, gemfibrozil, ibuprofen, naproxen, and propranolol) downregulated the expression of global regulatory genes (*korA*, *korB* and *tbrA*), resulting in the upregulation of RP4 transfer genes (Cen et al. 2020; Wang et al. 2021; He et al. 2022). Specifically, the expression of conjugative transfer transcriptional regulator genes, such as *traG* and *trbD*, mating pair apparatus genes, such as *trbA, trbK* and *trfA2*, and the replication regulator gene *traC1* were significantly upregulated in RP4 (Wang et al. 2021). The anti-cancer drug paclitaxel did not affect the expression of *korA* or *korB*, but instead upregulated the expression of *kilA* and *kilB* from RP4 (Yang et al. 2022). The *kilA* and *kilB* genes are host-lethal determinants repressed by *korA* and *korB*, respectively (Goncharoff et al. 1991). Mating pair apparatus genes (*traF* and *trbBp*) and DNA transfer and replication genes (*trfAp* and *traJ*) were upregulated with exposure to paclitaxel during conjugation (Yang et al. 2022). Accordingly, the authors suggested that the increased expression of *kilA* and *kilB* antagonized the functions of *korA* and *korB*, resulting in the de-repression of RP4 transfer genes (Thomson et al. 1993; Yang et al. 2022).

As a contact-dependent process, bacterial conjugation requires mating-pair formation between the donor and the recipient cells (Neil et al. 2021). Moreover, mating-pair stabilization is necessary for the donor and recipient cells to be in proximity long enough to enable successful plasmid transmission (Neil et al. 2021). These crucial steps are mediated by either adhesins displayed at the cell surface or conjugative pilus associated with the T4SS (Hospenthal et al. 2017). The T4SS-associated pilus also functions as a channel for the transfer of plasmid from the donor to the recipient cell (Samuels et al. 2000; Grohmann et al. 2018; Virolle et al. 2020). Unsurprisingly, some compounds that increased plasmid transmission also upregulated the expression of pilin-related genes in donor bacteria and conjugative plasmids. For example, carbamazepine exposure during conjugation significantly upregulated most pilin formation genes (*traA*, *traB*, *traH*, *traL*, and *traP*) on RP4 in donor cells (Wang et al. 2019). Likewise, another study reported that with exposure to diclofenac, gemfibrozil, ibuprofen, and naproxen, the expression of pilin-related genes on RP4 were significantly upregulated in donor cells (Wang et al. 2021). Artificial sweeteners that promoted RP4 transmission in *E. coli* also significantly increased the expression of several pilin formation genes (*traA*, *traB*, *traF,* and *traP)* and adhesion-relevant operons, when compared to untreated control (Yu et al. 2021). This could also enhance plasmid transmission by facilitating adhesion of donor and recipient cells (Hospenthal et al. 2017).

Antidepressants that promoted conjugative transfer of RP4 had differential effects on the expression of transfer genes. For example, while duloxetine and fluoxetine upregulated the expression of most transfer genes in RP4, bupropion and escitalopram exerted limited de-repression of transfer genes (Ding et al. 2022b). Sertraline upregulated the expression of RP4 transfer genes, including those involved in plasmid replication, pilus assembly, mating-pair formation and relaxasome assembly (Ding et al. 2022b), which correlated with sertraline having one of the highest conjugation enhancing effects out of the antidepressants.

### 6.6. Reactive oxygen species, oxidative stress, and the SOS response

Reactive oxygen species (ROS) are a natural by-product of bacterial metabolism. However, environmental stress can substantially increase production of intracellular ROS in bacteria and cause oxidative stress (Fasnacht and Polacek 2021). This activates bacterial SOS response, induces DNA repair and mutagenesis (McKenzie et al. 2000). The SOS increases genetic variability which improves the chances of survival for bacteria in stressful conditions (McKenzie et al. 2000). Accordingly, studies have suggested a link between the SOS response and the horizonal transfer of AMR genes (Beaber et al. 2004; Baharoglu et al. 2010; Mohanraj and Mandal 2022). Most of the compounds that enhanced plasmid transmission have also been reported to increase ROS production, induce oxidative stress, and upregulate the SOS response genes.

Clinically approved drugs (carbamazepine, diclofenac, gemfibrozil, ibuprofen, naproxen, and propranolol) that increased plasmid transmission elevated the levels of measured ROS in donor and recipient cells (Wang et al. 2019; 2021). This was accompanied by significant upregulation of ROS production-related genes, such as hydroperoxide reductase (*ahpC* and *ahpF*), oxidative demethylase (*alkB*), superoxide dismutase (*sodB* and *sodC*) and superoxide response (*soxS)* (Wang et al. 2019; 2021). Additionally, activities of the alkyl hydroperoxide reductase (AhpF) and superoxide dismutase (SodC) enzymes significantly increased when treated with the clinically approved drugs, which was reversed by the addition of the ROS scavenger thiourea. The addition of thiourea with the drugs reversed the ROS-mediated increase in plasmid transmission, suggesting increased ROS plays an important role increasing plasmid transmission (Wang et al. 2021).

Antidepressants duloxetine, fluoxetine, and sertraline promoted conjugation by triggering ROS overproduction in donor and recipient cells, whereas bupropion promoted plasmid transmission without inducing ROS (Ding et al. 2022b). Consequently, addition of thiourea during conjugation reversed the ROS-mediated elevated plasmid transmission by duloxetine, fluoxetine, and sertraline, but not bupropion. The authors suggested that the conjugation promoting effect of bupropion was primarily mediated by increased membrane permeability rather than ROS overproduction (Ding et al. 2022b). Paracetamol is another clinically approved drug that has been reported by two different studies to cause ROS overproduction in both donor and recipient cells (Jia et al. 2021; Cui et al. 2022).

Increased ROS production in donor and recipient bacteria is also implicated in enhanced plasmid transfer by environmental pollutants, including disinfection by-products, the herbicide glyphosate, and heavy metals (Zhang et al. 2018; 2021; He et al. 2022). Sodium nitroprusside does not affect the expression of genes involved in the SOS response, but instead upregulates the nitrative stress response genes (Huang et al. 2022). The authors suggested that NO boosted plasmid transmission through other mechanisms rather than the widely reported SOS response (Huang et al. 2022). The food preservatives sodium nitrite, sodium benzoate, and triclocarban also do not affect ROS production (Cen et al. 2020). However, the three compounds significantly upregulated the expression of oxidative stress and SOS response-related genes, and the stress-response sigma factor gene *rpoS* (Cen et al. 2020). This suggests with exposure to the food preservatives, induction of the RpoS regulon and the SOS response plays a bigger role in enhancing plasmid transmission than ROS production.

It is likely that environmental stresses, trigger ROS production and/or the SOS response may facilitate conjugation. Accordingly, a sublethal intensity of electrical current increased the conjugative transfer of pKJK5 in *P. putida* by inducing ROS generation and overexpression of core oxidative stress genes (Li et al. 2023a).

### 6.7. Changes in the intracellular metabolite levels

A recent study by Huang et al. reported that sodium nitroprusside promoted plasmid transmission by affecting the transcription of metabolism genes (Huang et al. 2022). The *mtr* gene encoding the tryptophan-specific transport protein was downregulated and HPLC analysis confirmed in both donor and recipient the intracellular concentration of tryptophan was significantly elevated. Conversely, intracellular levels of methionine were significantly reduced with exposure to sodium nitroprusside due to the upregulation of genes involved in the catabolism and transport of sulfur containing amino acids. Interestingly, the exogenous addition of tryptophan into the conjugation system caused a further downregulation of *korA* and *korB* genes than sodium nitroprusside alone. Furthermore, the corresponding expression of transfer genes were dramatically upregulated. Conversely, exogenous addition of methionine was found to inhibit the NO-mediated repression of *korA* and *korB* genes, resulting in the downregulation of transfer genes. This suggests the increased tryptophan and reduced methionine levels induced by the NO donor sodium nitroprusside contributes to enhanced plasmid transmission. This unique mechanism has not been reported with any of the other compounds discussed in this review.

Plasmid acquisition and carriage alters different cellular processes within the host cell (Billane et al. 2022). Acquisition of several plasmids in *P. aeruginosa*, including the conjugative IncP-1β plasmid pAKD1, significantly affected the expression of several chromosomal genes encoding proteins involved in amino acid metabolism, such as aspartate-semialdehyde dehydrogenase and glutamine synthetase (San Millan et al. 2018). A recent study reported *Haemophilus influenzae* carrying the mobilizable ColE1 plasmid pB1000 displayed changes in amino acid metabolism to compensate for plasmid carriage (Ares-Arroyo et al. 2022). Specifically, genes involved in tryptophan transport were downregulated and genes involved in tryptophan biosynthesis were upregulated (Ares-Arroyo et al. 2022), suggesting increased intracellular pool of tryptophan. Genes involved in the catabolism and transport of sulfur containing amino acids was downregulated or mutated (Ares-Arroyo et al. 2022), suggesting reduced intracellular levels of methionine and serine. Therefore, changes in amino acid metabolism could be involved in plasmid compensation to ameliorate or overcome the fitness cost of plasmid carriage, thereby increasing the number of transconjugant bacteria (Billane et al. 2022).

## 7. What level of risk do non-antibiotic pharmaceuticals and environmental pollutants pose to the spread of AMR plasmids?

The potential level of risk posed by non-antibiotic pharmaceuticals and environmental pollutants in the dissemination of AMR plasmids is dependent on their effect concentration, range of activity and mechanism of action (Table S3 and S4). Non-antibiotic pharmaceuticals, including antidepressants (escitalopram, fluoxetine, and sertraline), the anti-epileptic drug carbamazepine, and non-steroidal anti-inflammatory drugs (diclofenac and ibuprofen), promoted plasmid transfer (Wang et al. 2019; 2021; 2022; Ding et al. 2022b) and induced antibiotic persistence/tolerance in bacteria at clinically relevant concentrations through similar mechanisms, such as ROS overproduction, activation of SOS response, and cell membrane damage (Wang et al. 2020e; Ou et al. 2022; Wang et al. 2023; Li et al. 2023b). Artificial sweeteners at recommended daily use concentrations also promoted conjugative transfer of plasmids in the mouse gut microbiota (Yu et al. 2023). Therefore, such compounds are likely to pose a high risk in spreading AMR genes in clinical or daily settings (Table S3). On the other hand, drugs like paracetamol increased conjugative transfer of AMR plasmids at concentrations that are significantly higher than clinically and environmentally relevant concentrations (Jia et al. 2021), suggesting a lower level of risk (Table S3).

Despite being classified as non-antibiotic pharmaceuticals, most high-risk compounds that promoted transfer of AMR plasmids also possess a degree of antimicrobial activity. For example, sertraline and fluoxetine inhibited growth of some gut microbiota-associated and pathogenic bacteria at clinically relevant concentrations (Munoz-Bellido et al. 2000; McGovern et al. 2019; Rukavishnikov et al. 2023). Similarly, ibuprofen also inhibited growth of cystic fibrosis-associated Gram-negative pathogens (Shah et al. 2018) and diclofenac had antibacterial activity against several different Gram-positive and Gram-negative pathogens (Dutta et al. 2007). Therefore, the potential risk posed by non-antibiotic pharmaceuticals in the dissemination of AMR plasmids could be predicted based on their antimicrobial activity and ability to trigger stress responses in bacteria.

Environmental pollutants, including herbicides (glyphosate), disinfection-by-products, nanomaterials (nanoalumina), and heavy metals (arsenic, copper, lead, and mercury) are likely to pose a high level of risk for disseminating AMR genes because they increased conjugative transfer of AMR plasmids at very low concentrations that have been reported in the environment (Table S4). The co-location of heavy-metal resistance and AMR genes on plasmids further increases the risk of these compounds increasing transmission, as they can provide a selective advantage for bacteria (Vats et al. 2022). Certain emerging pollutants such as ionic liquids and some metallic nanomaterials are not currently used widely so there is insufficient data on their environmental presence. However, based on their ability to promote conjugative transfer of AMR plasmids, their environmental footprint should be closely monitored. In addition, global locations associated with high levels of AMR genes and high levels of pollution should be of particular concern. To date there is insufficient data to determine if high levels of pollution in such locations has been one of the contributing factors to high levels of AMR. But future research should take into consideration the potential of such pollutants to drive AMR transmission within bacteria.

## 8. Conclusion

The injudicious use and over consumption of antibiotics is a well-established driver of the increasing global rates of AMR (Klein et al. 2018). However, it is becoming increasingly evident that commonly used non-antibiotic compounds have a greater impact on AMR than previously thought. As discussed in this review, a wide range of non-antibiotic pharmaceuticals, several food additives, and various environmental pollutants have been demonstrated to promote the transmission of various conjugative plasmids. However, most of the studies discussed in this review were conducted using laboratory-adapted *E. coli* strains (e.g. *E. coli* J53) and model plasmids (e.g. RP4 and RK2). Therefore, the impact of most non-antibiotic compounds on the transmission of AMR plasmids in clinically relevant isolates and plasmids remains to be investigated. Furthermore, there is no data on the influence of non-antibiotic pharmaceuticals on plasmid transmission *in vivo* where the conditions experienced by bacteria are substantially different (Neil et al. 2021). Nonetheless, certain compounds like naproxen, paracetamol, and sertraline increased conjugative transfer of plasmids carrying clinically important antibiotic resistance genes (*aacA4*, *bla*_NDM-1_, *mcr*-1 and *tet*(X4)), which shows that these compounds could pose a risk for disseminating antibiotic resistance in clinical settings. However, for paracetamol the concentration tested was above the maximum serum concentration. The risk posed is likely to be higher with antidepressants, which have been recently shown to induce resistance mutations and enhance persistence to multiple antibiotics at clinically relevant concentrations and exposure times (Wang et al. 2023).

The compounds discussed in this review had common mechanisms of enhancing plasmid transmission. These included increased membrane permeability, ROS overproduction, and upregulation of SOS response genes in both donor and recipient cells. In donor cells, transfer genes on conjugative plasmids were often upregulated. Other mechanisms such as altered metabolite levels remain elusive and requires further investigation. It is evident from these studies that non-antibiotic compounds have a wide range of genetic and physiological effects on bacteria. Therefore, it is likely that these compounds increase plasmid transmission through several different mechanisms simultaneously rather than a single mechanism alone.

## 9. Outlook and future perspectives

Compared to our knowledge of the relationship between antibiotic use and AMR, the link between non-antibiotic compounds and AMR is poorly understood. Most studies that have suggested a role for non-antibiotic pharmaceuticals in promoting horizontal gene transfer are recent (Wang et al. 2021; 2022; Ding et al. 2022b). Hence, we anticipate that further studies will investigate the impact of other commonly used pharmaceuticals and compounds on horizontal transfer of AMR genes and use real-world (clinical or environmental) plasmids to test their effects. This could support clinicians to make more informed decisions regarding prescribing of drugs to patients. For example, if a certain drug is known to promote the transmission of AMR plasmids, it could exacerbate an existing AMR infection by disseminating said plasmids to other bacteria within the patient. Likewise, a similar drug that has minimal or no impact on plasmid transfer could be the preferred drug of choice for the patient.

The studies discussed in this review were mostly carried out using standard laboratory strains and model conjugative plasmids. Therefore, the effect of non-antibiotic compounds on the horizontal transfer of AMR genes needs to be investigated using current clinically relevant strains and plasmids. This would better reflect the influence of said drugs on the transfer of clinically relevant plasmids in problematic pathogens. Consequently, the impact of using non-antibiotic pharmaceuticals in hospitals and clinical settings on existing AMR infections remains to be investigated. If a particular drug is identified as a potential driver of promoting transmission of AMR plasmids, its use could be more closely monitored and where possible alternative drugs could be used.

Noticeably, there is a lack of data on the impact of non-antibiotic compounds on conjugative plasmid transfer in Gram-positive bacteria. Plasmid mediated antibiotic resistance is also a significant problem in clinically important Gram-positive pathogens, such as *Enterococcus faecium* and *Staphylococcus aureus* (Kohler et al. 2019). Furthermore, Gram-positive bacteria are important reservoirs of AMR genes in the gut microbiota (Sommer et al. 2009; McInnes et al. 2020). Hence, the impact of non-antibiotic compounds associated with humans and the environment on horizontal transfer of AMR genes need be investigated in Gram-positive bacteria. There is also a lack of information on compounds which may impact rates of transduction and its contribution to HGT, an area which needs further study.

Antibiotic use is known to affect the human microbiome and host health (Patangia et al. 2022). However, it is becoming increasingly evident that commonly used drugs and food additives can also impact the composition and metabolic function of the gut microbiota (Walsh et al. 2018; Brusselaers 2019; Vich Vila et al. 2020; Weersma et al. 2020; Chassaing et al. 2022). The gut microbiome has been described as a hotspot for the horizontal transfer of AMR genes due to the high density of micro-organisms (Penders et al. 2013; McInnes et al. 2020; Kessler et al. 2023). Thus, the influence of non-antibiotic pharmaceuticals and food additives on the horizontal transfer of AMR genes in the gut microbiota remains unknown and warrants further investigation. Ideally, *in vitro,* and *in vivo* models of bacterial conjugation that closely recapitulate the gut microbiome should be used (Ott and Mellata 2022; Kessler et al. 2023).

The presence of pollutants in the environment can have a significant impact on microbial communities (Chen et al. 2019). However, this relationship is complex and can be affected by several different factors, such as the type of environment, the pollutant load, the origin of the pollutant, and the microbial community of the environment (Kraemer et al. 2019). As discussed in this review, there is evidence to suggest that pollutants at environmentally relevant concentrations can affect plasmid transmission. However, most of these studies were conducted under laboratory conditions. Therefore, the impact of pollutants needs to be investigated using models that better reflect the microbial communities present in different environmental niches (Sørensen et al. 2005; Li et al. 2019). The presence of these pollutants in the environment could also facilitate the spread of AMR plasmids in environmental bacteria, which could be passed along the food chain to animals and humans (Larsson and Flach 2022). Thus, AMR is yet another reason why policymakers should act to closely monitor and regulate the disposal of non-antibiotic drugs and other industrial sources of waste into the environment.

Based on the mechanism of how non-antibiotic compounds promote plasmid transmission, there are several potential strategies to inhibit or reduce plasmid transmission. Targeting the conjugation apparatus is a viable strategy to inhibit bacterial conjugation. Previous studies have shown that compounds that target the components of the T4SS apparatus are effective inhibitors of bacterial conjugation (Garcillan-Barcia et al. 2007; Casu et al. 2016; Getino et al. 2016; Ripoll-Rozada et al. 2016; Garcia-Cazorla et al. 2018). Yet, there are no anti-plasmid compounds in clinical development. With the increasing availability of high-resolution structures of the various T4SSs (Low et al. 2014; Liu et al. 2022; Macé et al. 2022), we anticipate that further promising T4SS inhibitors will be identified through structure-activity relationship. Increased understanding of the conjugation process and the structure of the T4SS apparatus could present new drug targets for the development of anti-plasmid compounds.

## Supplementary Material

Supplemental Material

## References

[CIT0001] Acman M, Wang R, van Dorp L, Shaw LP, Wang Q, Luhmann N, Yin Y, Sun S, Chen H, Wang H, et al. 2022. Role of mobile genetic elements in the global dissemination of the carbapenem resistance gene *bla*NDM. Nat Commun. 13(1):1131. doi:10.1038/s41467-022-28819-2. 35241674 PMC8894482

[CIT0002] Ahmed MAE-GE-S, Yang Y, Yang Y, Yan B, Chen G, Hassan RM, Zhong L-L, Chen Y, Roberts AP, Wu Y, et al. 2021. Emergence of hypervirulent carbapenem-resistant *Klebsiella pneumoniae* coharboring a *bla*(NDM-1)-carrying virulent plasmid and a *bla*(KPC-2)-carrying plasmid in an Egyptian Hospital. mSphere. 6(3):1–6. doi:10.1128/mSphere.00088-21. PMC8265623 34011682

[CIT0003] Al-Tohamy R, Ali SS, Li F, Okasha KM, Mahmoud YAG, Elsamahy T, Jiao H, Fu Y, Sun J. 2022. A critical review on the treatment of dye-containing wastewater: ecotoxicological and health concerns of textile dyes and possible remediation approaches for environmental safety. Ecotoxicol Environ Saf. 231:113160. doi:10.1016/j.ecoenv.2021.113160. 35026583

[CIT0004] Alderton I, Palmer BR, Heinemann JA, Pattis I, Weaver L, Gutiérrez-Ginés MJ, Horswell J, Tremblay LA. 2021. The role of emerging organic contaminants in the development of antimicrobial resistance. Emerg Contaminants. 7:160–171. doi:10.1016/j.emcon.2021.07.001.

[CIT0005] Arcari G, Carattoli A. 2022. Global spread and evolutionary convergence of multidrug-resistant and hypervirulent *Klebsiella pneumoniae* high-risk clones. Pathog Glob Health. 117(4):328–341. 36089853 10.1080/20477724.2022.2121362 PMC10177687

[CIT0006] Ares-Arroyo M, Fernandez-Garcia M, Wedel E, Montero N, Barbas C, Rey-Stolle MF, Garcia A, Gonzalez-Zorn B. 2022. Genomics, transcriptomics, and metabolomics reveal that minimal modifications in the host are crucial for the compensatory evolution of ColE1-like plasmids. mSphere. 7(6):e0018422. doi:10.1128/msphere.00184-22. 36416553 PMC9769657

[CIT0007] Arias-Andres M, Klumper U, Rojas-Jimenez K, Grossart HP. 2018. Microplastic pollution increases gene exchange in aquatic ecosystems. Environ Pollut. 237:253–261. doi:10.1016/j.envpol.2018.02.058. 29494919

[CIT0008] Baharoglu Z, Bikard D, Mazel D. 2010. Conjugative DNA transfer induces the bacterial SOS response and promotes antibiotic resistance development through integron activation. PLoS Genet. 6(10):e1001165. doi:10.1371/journal.pgen.1001165. 20975940 PMC2958807

[CIT0009] Baig N, Kammakakam I, Falath W. 2021. Nanomaterials: a review of synthesis methods, properties, recent progress, and challenges. Mater Adv. 2(6):1821–1871. doi:10.1039/D0MA00807A.

[CIT0010] Bandyopadhyay A, Ghoshal S, Mukherjee A. 2008. Genotoxicity testing of low-calorie sweeteners: aspartame, acesulfame-K, and saccharin. Drug Chem Toxicol. 31(4):447–457. doi:10.1080/01480540802390270. 18850355

[CIT0011] Beaber JW, Hochhut B, Waldor MK. 2004. SOS response promotes horizontal dissemination of antibiotic resistance genes. Nature. 427(6969):72–74. doi:10.1038/nature02241. 14688795

[CIT0012] Berge C, Waksman G, Terradot L. 2017. Structural and molecular biology of type IV secretion systems. Curr Top Microbiol Immunol. 413:31–60. doi:10.1007/978-3-319-75241-9_2. 29536354

[CIT0013] Bertani B, Ruiz N. 2018. Function and biogenesis of lipopolysaccharides. EcoSal plus. 8(1):1–19. doi:10.1128/ecosalplus.ESP-0001-2018. PMC6091223 30066669

[CIT0014] Biedrzycka M, Izdebski R, Urbanowicz P, Polańska M, Hryniewicz W, Gniadkowski M, Literacka E. 2022. MDR carbapenemase-producing *Klebsiella pneumoniae* of the hypervirulence-associated ST23 clone in Poland, 2009-19. J Antimicrob Chemother. 77(12):3367–3375. doi:10.1093/jac/dkac326. 36177793

[CIT0015] Billane K, Harrison E, Cameron D, Brockhurst MA. 2022. Why do plasmids manipulate the expression of bacterial phenotypes? Philos Trans R Soc Lond B Biol Sci. 377(1842):20200461. doi:10.1098/rstb.2020.0461. 34839708 PMC8628079

[CIT0016] Bouet JY, Funnell BE. 2019. Plasmid localization and partition in Enterobacteriaceae. EcoSal plus. 8(2):1–23. doi:10.1128/ecosalplus.ESP-0003-2019. PMC11573283 31187729

[CIT0017] Brett CN, Barnett SG, Pearson J. 2012. Postoperative plasma paracetamol levels following oral or intravenous paracetamol administration: a double-blind randomised controlled trial. Anaesth Intensive Care. 40(1):166–171. doi:10.1177/0310057X1204000121. 22313079

[CIT0018] Brusselaers N. 2019. Prescribed drugs and the microbiome. Gastroenterol Clin North Am. 48(3):331–342. doi:10.1016/j.gtc.2019.04.002. 31383274

[CIT0019] Buberg ML, Witso IL, L’Abee-Lund TM, Wasteson Y. 2020. Zinc and copper reduce conjugative transfer of resistance plasmids from extended-spectrum beta-lactamase-producing *Escherichia coli*. Microb Drug Resist. 26(7):842–849. doi:10.1089/mdr.2019.0388. 31951514

[CIT0020] Buckner MMC, Ciusa ML, Piddock LJV. 2018. Strategies to combat antimicrobial resistance: anti-plasmid and plasmid curing. FEMS Microbiol Rev. 42(6):781–804. doi:10.1093/femsre/fuy031. 30085063 PMC6199537

[CIT0021] Buckner MMC, Ciusa ML, Meek RW, Moorey AR, McCallum GE, Prentice EL, Reid JP, Alderwick LJ, Di Maio A, Piddock LJV. 2020. HIV drugs inhibit transfer of plasmids carrying extended-spectrum beta-lactamase and carbapenemase genes. mBio. 11(1):1–18. doi:10.1128/mBio.03355-19. PMC7042701 32098822

[CIT0022] Cabezon E, Ripoll-Rozada J, Pena A, de la Cruz F, Arechaga I. 2015. Towards an integrated model of bacterial conjugation. FEMS Microbiol Rev. 39(1):81–95. doi:10.1111/1574-6976.12085. 25154632

[CIT0023] Camargo JF, Simkins J, Beduschi T, Tekin A, Aragon L, Pérez-Cardona A, Prado CE, Morris MI, Abbo LM, Cantón R. 2015. Successful treatment of carbapenemase-producing pandrug-resistant *Klebsiella pneumoniae* bacteremia. Antimicrob Agents Chemother. 59(10):5903–5908. doi:10.1128/AAC.00655-15. 26386029 PMC4576028

[CIT0024] Casu B, Smart J, Hancock MA, Smith M, Sygusch J, Baron C. 2016. Structural analysis and inhibition of TraE from the pKM101 type IV secretion system. J Biol Chem. 291(45):23817–23829. doi:10.1074/jbc.M116.753327. 27634044 PMC5095433

[CIT0025] Cen T, Zhang X, Xie S, Li D. 2020. Preservatives accelerate the horizontal transfer of plasmid-mediated antimicrobial resistance genes via differential mechanisms. Environ Int. 138:105544. doi:10.1016/j.envint.2020.105544. 32172042

[CIT0026] Chassaing B, Compher C, Bonhomme B, Liu Q, Tian Y, Walters W, Nessel L, Delaroque C, Hao F, Gershuni V, et al. 2022. Randomized controlled-feeding study of dietary emulsifier carboxymethylcellulose reveals detrimental impacts on the gut microbiota and metabolome. Gastroenterology. 162(3):743–756. doi:10.1053/j.gastro.2021.11.006. 34774538 PMC9639366

[CIT0027] Chen J, McIlroy SE, Archana A, Baker DM, Panagiotou G. 2019. A pollution gradient contributes to the taxonomic, functional, and resistome diversity of microbial communities in marine sediments. Microbiome. 7(1):104. doi:10.1186/s40168-019-0714-6. 31307536 PMC6632204

[CIT0028] Chow LKM, Ghaly TM, Gillings MR. 2021. A survey of sub-inhibitory concentrations of antibiotics in the environment. J Environ Sci (China). 99:21–27. doi:10.1016/j.jes.2020.05.030. 33183698

[CIT0029] Cousins IT, Ng CA, Wang Z, Scheringer M. 2019. Why is high persistence alone a major cause of concern? Environ Sci Process Impacts. 21(5):781–792. doi:10.1039/c8em00515j. 30973570

[CIT0030] Cui Y, Gao J, Guo Y, Li Z, Wang Z, Zhao Y. 2022. Unraveling the impact and mechanism of antipyretic paracetamol on intergenera conjugative plasmid transfer. Environ Res. 215:114263. doi:10.1016/j.envres.2022.114263. 36075475

[CIT0031] Darby EM, Trampari E, Siasat P, Gaya MS, Alav I, Webber MA, Blair JMA. 2023. Molecular mechanisms of antibiotic resistance revisited. Nat Rev Microbiol. 21(5):280–295. doi:10.1038/s41579-022-00820-y. 36411397

[CIT0032] Davies NM, Anderson KE. 1997. Clinical pharmacokinetics of naproxen. Clin Pharmacokinet. 32(4):268–293. doi:10.2165/00003088-199732040-00002. 9113437

[CIT0033] Dimitriu T. 2022. Evolution of horizontal transmission in antimicrobial resistance plasmids. Microbiology (Reading). 168(7):1–9. doi:10.1099/mic.0.001214. 35849537

[CIT0034] Ding M, Ye Z, Liu L, Wang W, Chen Q, Zhang F, Wang Y, Sjöling Å, Martín-Rodríguez AJ, Hu R, et al. 2022a. Subinhibitory concentration antibiotic promotes horizontal transfer of plasmid-borne multi-antibiotic resistance genes with *Klebsiella pneumonia* and *E. coli*. Front Microbiol. 13:1–11. doi:10.3389/fmicb.2022.1017092. PMC9678054 36419429

[CIT0035] Ding P, Lu J, Wang Y, Schembri MA, Guo J. 2022b. Antidepressants promote the spread of antibiotic resistance via horizontally conjugative gene transfer. Environmental Microbiology n/a. 24(11):5261–5276. doi:10.1111/1462-2920.16165. PMC9804927 36054646

[CIT0036] Doi Y. 2019. Treatment options for carbapenem-resistant Gram-negative bacterial infections. Clin Infect Dis. 69(Suppl 7):S565–S575. doi:10.1093/cid/ciz830. 31724043 PMC6853760

[CIT0037] Duan W, Meng F, Wang F, Liu Q. 2017. Environmental behavior and eco-toxicity of xylene in aquatic environments: a review. Ecotoxicol Environ Saf. 145:324–332. doi:10.1016/j.ecoenv.2017.07.050. 28756253

[CIT0038] Duke J, Guiney DG. Jr 1983. The role of lipopolysaccharide structure in the recipient cell during plasmid-mediated bacterial conjugation. Plasmid. 9(2):222–226. doi:10.1016/0147-619x(83)90024-0. 6856692

[CIT0039] Dutta NK, Annadurai S, Mazumdar K, Dastidar SG, Kristiansen JE, Molnar J, Martins M, Amaral L. 2007. Potential management of resistant microbial infections with a novel non-antibiotic: the anti-inflammatory drug diclofenac sodium. Int J Antimicrob Agents. 30(3):242–249. doi:10.1016/j.ijantimicag.2007.04.018. 17644318

[CIT0040] Element SJ, Moran RA, Beattie E, Hall RJ, van Schaik W, Buckner MMC. 2023. Growth in a biofilm promotes conjugation of a *bla*NDM-1-bearing plasmid between *Klebsiella pneumoniae* strains. *bioRxiv* 2023.2001.2005.522703. 10.1128/msphere.00170-23 PMC10449501 37417759

[CIT0041] Fasnacht M, Polacek N. 2021. Oxidative stress in bacteria and the central dogma of molecular biology. Front Mol Biosci. 8:671037. doi:10.3389/fmolb.2021.671037. 34041267 PMC8141631

[CIT0042] Feng M, Ye C, Zhang S, Sharma VK, Manoli K, Yu X. 2022. Bisphenols promote the conjugative transfer of antibiotic resistance genes without damaging cell membrane. Environ Chem Lett. 20(3):1553–1560. doi:10.1007/s10311-022-01397-x.

[CIT0043] Fowler D, Coyle M, Skiba U, Sutton MA, Cape JN, Reis S, Sheppard LJ, Jenkins A, Grizzetti B, Galloway JN, et al. 2013. The global nitrogen cycle in the twenty-first century. Philos Trans R Soc Lond B Biol Sci. 368(1621):20130164. doi:10.1098/rstb.2013.0164. 23713126 PMC3682748

[CIT0044] Garcia-Cazorla Y, Getino M, Sanabria-Rios DJ, Carballeira NM, de la Cruz F, Arechaga I, Cabezon E. 2018. Conjugation inhibitors compete with palmitic acid for binding to the conjugative traffic ATPase TrwD, providing a mechanism to inhibit bacterial conjugation. J Biol Chem. 293(43):16923–16930. doi:10.1074/jbc.RA118.004716. 30201608 PMC6204903

[CIT0045] Garcillan-Barcia MP, Jurado P, Gonzalez-Perez B, Moncalian G, Fernandez LA, de la Cruz F. 2007. Conjugative transfer can be inhibited by blocking relaxase activity within recipient cells with intrabodies. Mol Microbiol. 63(2):404–416. doi:10.1111/j.1365-2958.2006.05523.x. 17163977

[CIT0046] Getino M, de la Cruz F. 2018. Natural and artificial strategies to control the conjugative transmission of plasmids. Microbiol Spectr. 6(1):1–25. doi:10.1128/microbiolspec.MTBP-0015-2016. PMC11633558 29327679

[CIT0047] Getino M, Fernandez-Lopez R, Palencia-Gandara C, Campos-Gomez J, Sanchez-Lopez JM, Martinez M, Fernandez A, de la Cruz F. 2016. Tanzawaic acids, a chemically novel set of bacterial conjugation inhibitors. PLoS One. 11(1):e0148098. doi:10.1371/journal.pone.0148098. 26812051 PMC4727781

[CIT0048] Gillezeau C, van Gerwen M, Shaffer RM, Rana I, Zhang L, Sheppard L, Taioli E. 2019. The evidence of human exposure to glyphosate: a review. Environ Health. 18(1):2. doi:10.1186/s12940-018-0435-5. 30612564 PMC6322310

[CIT0049] Gomes IB, Maillard J-Y, Simões LC, Simões M. 2020. Emerging contaminants affect the microbiome of water systems—strategies for their mitigation. Npj Clean Water. 3(1):39. doi:10.1038/s41545-020-00086-y.

[CIT0050] Goncharoff P, Saadi S, Chang CH, Saltman LH, Figurski DH. 1991. Structural, molecular, and genetic analysis of the *kilA* operon of broad-host-range plasmid RK2. J Bacteriol. 173(11):3463–3477. doi:10.1128/jb.173.11.3463-3477.1991. 2045366 PMC207960

[CIT0051] Grohmann E, Christie PJ, Waksman G, Backert S. 2018. Type IV secretion in Gram-negative and Gram-positive bacteria. Mol Microbiol. 107(4):455–471. doi:10.1111/mmi.13896. 29235173 PMC5796862

[CIT0052] Gu D, Dong N, Zheng Z, Lin D, Huang M, Wang L, Chan EW-C, Shu L, Yu J, Zhang R, et al. 2018. A fatal outbreak of ST11 carbapenem-resistant hypervirulent *Klebsiella pneumoniae* in a Chinese hospital: a molecular epidemiological study. Lancet Infect Dis. 18(1):37–46. doi:10.1016/S1473-3099(17)30489-9. 28864030

[CIT0053] Gullberg E, Cao S, Berg OG, Ilbäck C, Sandegren L, Hughes D, Andersson DI. 2011. Selection of resistant bacteria at very low antibiotic concentrations. PLoS Pathog. 7(7):e1002158. doi:10.1371/journal.ppat.1002158. 21811410 PMC3141051

[CIT0054] He K, Xue B, Yang X, Wang S, Li C, Zhang X, Zhao C, Wang X, Qiu Z, Shen Z, et al. 2022. Low-concentration of trichloromethane and dichloroacetonitrile promote the plasmid-mediated horizontal transfer of antibiotic resistance genes. J Hazard Mater. 425:128030. doi:10.1016/j.jhazmat.2021.128030. 34986571

[CIT0055] Helinski DR. 2022. A brief history of plasmids. EcoSal Plus. 10(1):eESP00282021. doi:10.1128/ecosalplus.ESP-0028-2021. 35373578 PMC10729939

[CIT0056] Hennequin C, Aumeran C, Robin F, Traore O, Forestier C. 2012. Antibiotic resistance and plasmid transfer capacity in biofilm formed with a CTX-M-15-producing *Klebsiella pneumoniae* isolate. J Antimicrob Chemother. 67(9):2123–2130. doi:10.1093/jac/dks169. 22577106

[CIT0057] Hoekstra WPM, Havekes AM. 1979. On the role of the recipient cell during conjugation in *Escherichia coli*. Antonie Van Leeuwenhoek. 45(1):13–18. doi:10.1007/BF00400773. 45216

[CIT0058] Hooper DC, Wolfson JS, McHugh GL, Swartz MD, Tung C, Swartz MN. 1984. Elimination of plasmid pMG110 from *Escherichia coli* by novobiocin and other inhibitors of DNA gyrase. Antimicrob Agents Chemother. 25(5):586–590. doi:10.1128/AAC.25.5.586. 6329090 PMC185592

[CIT0059] Hospenthal MK, Costa TRD, Waksman G. 2017. A comprehensive guide to pilus biogenesis in Gram-negative bacteria. Nat Rev Microbiol. 15(6):365–379. doi:10.1038/nrmicro.2017.40. 28496159

[CIT0060] Huang H, Feng G, Wang M, Liu C, Wu Y, Dong L, Feng L, Zheng X, Chen Y. 2022. Nitric oxide: a neglected driver for the conjugative transfer of antibiotic resistance genes among wastewater microbiota. Environ Sci Technol. 56(10):6466–6478. doi:10.1021/acs.est.2c01889. 35512279

[CIT0061] Huang YH, Chou SH, Liang SW, Ni CE, Lin YT, Huang YW, Yang TC. 2018. Emergence of an XDR and carbapenemase-producing hypervirulent *Klebsiella pneumoniae* strain in Taiwan. J Antimicrob Chemother. 73(8):2039–2046. doi:10.1093/jac/dky164. 29800340

[CIT0062] Hunger M, Schmucker R, Kishan V, Hillen W. 1990. Analysis and nucleotide sequence of an origin of DNA replication in *Acinetobacter calcoaceticus* and its use for *Escherichia coli* shuttle plasmids. Gene. 87(1):45–51. doi:10.1016/0378-1119(90)90494-c. 2185139

[CIT0063] Ilangovan A, Connery S, Waksman G. 2015. Structural biology of the Gram-negative bacterial conjugation systems. Trends Microbiol. 23(5):301–310. doi:10.1016/j.tim.2015.02.012. 25825348

[CIT0064] Jia X, Zhu Y, Jia P, Liu X, Yu W, Li X, Xu Y, Yang Q. 2022. Emergence of a superplasmid coharboring hypervirulence and multidrug resistance genes in *Klebsiella pneumoniae* poses new challenges to public health. Microbiol Spectr. 10(6):e0263422. doi:10.1128/spectrum.02634-22. 36264236 PMC9769819

[CIT0065] Jia Y, Wang Z, Fang D, Yang B, Li R, Liu Y. 2021. Acetaminophen promotes horizontal transfer of plasmid-borne multiple antibiotic resistance genes. Sci Total Environ. 782:146916. doi:10.1016/j.scitotenv.2021.146916.

[CIT0066] Jiao YN, Chen H, Gao RX, Zhu YG, Rensing C. 2017. Organic compounds stimulate horizontal transfer of antibiotic resistance genes in mixed wastewater treatment systems. Chemosphere. 184:53–61. doi:10.1016/j.chemosphere.2017.05.149. 28578196

[CIT0067] Jin M, Yuan H, Liu B, Peng J, Xu L, Yang D. 2020. Review of the distribution and detection methods of heavy metals in the environment. Anal Methods. 12(48):5747–5766. doi:10.1039/d0ay01577f. 33231592

[CIT0068] Johnson AP, Woodford N. 2013. Global spread of antibiotic resistance: the example of New Delhi metallo-beta-lactamase (NDM)-mediated carbapenem resistance. J Med Microbiol. 62(Pt 4):499–513. doi:10.1099/jmm.0.052555-0. 23329317

[CIT0069] Johnston C, Martin B, Fichant G, Polard P, Claverys J-P. 2014. Bacterial transformation: distribution, shared mechanisms and divergent control. Nat Rev Microbiol. 12(3):181–196. doi:10.1038/nrmicro3199. 24509783

[CIT0070] Joint Formulary Committee. 2022. British National Formulary (BNF84). UK: Pharmaceutical Press.

[CIT0071] Jurėnas D, Fraikin N, Goormaghtigh F, Van Melderen L. 2022. Biology and evolution of bacterial toxin–antitoxin systems. Nat Rev Microbiol. 20(6):335–350. doi:10.1038/s41579-021-00661-1. 34975154

[CIT0072] Kaur G, Kumar H, Singla M. 2022. Diverse applications of ionic liquids: a comprehensive review. J Mol Liquids. 351:118556. doi:10.1016/j.molliq.2022.118556.

[CIT0073] Kessler C, Hou J, Neo O, Buckner MMC. 2023. *In situ*, *in vivo*, and *in vitro* approaches for studying AMR plasmid conjugation in the gut microbiome. FEMS Microbiology Reviews. 47(1):1–13. doi:10.1093/femsre/fuac044. PMC9841969 36341518

[CIT0074] Klein EY, Van Boeckel TP, Martinez EM, Pant S, Gandra S, Levin SA, Goossens H, Laxminarayan R. 2018. Global increase and geographic convergence in antibiotic consumption between 2000 and 2015. Proc Natl Acad Sci U S A. 115(15):E3463–E3470. doi:10.1073/pnas.1717295115. 29581252 PMC5899442

[CIT0075] Kohler V, Keller W, Grohmann E. 2019. Regulation of gram-positive conjugation. Front Microbiol. 10:1134. doi:10.3389/fmicb.2019.01134. 31191478 PMC6540685

[CIT0076] Kopotsa K, Osei Sekyere J, Mbelle NM. 2019. Plasmid evolution in carbapenemase-producing Enterobacteriaceae: a review. Ann N Y Acad Sci. 1457(1):61–91. doi:10.1111/nyas.14223. 31469443

[CIT0077] Koraimann G. 2018. Spread and persistence of virulence and antibiotic resistance genes: a ride on the F plasmid conjugation module. EcoSal plus. 8(1):1–23. doi:10.1128/ecosalplus.ESP-0003-2018. PMC11575672 30022749

[CIT0078] Kothari A, Kumar P, Gaurav A, Kaushal K, Pandey A, Yadav SRM, Jain N, Omar BJ. 2023. Association of antibiotics and heavy metal arsenic to horizontal gene transfer from multidrug-resistant clinical strains to antibiotic-sensitive environmental strains. J Hazard Mater. 443(Pt B):130260. doi:10.1016/j.jhazmat.2022.130260. 36327832

[CIT0079] Kraemer SA, Ramachandran A, Perron GG. 2019. Antibiotic pollution in the environment: from microbial ecology to public policy. Microorganisms. 7(6):180. doi:10.3390/microorganisms7060180. 31234491 PMC6616856

[CIT0080] Krasner SW. 2009. The formation and control of emerging disinfection by-products of health concern. Philos Trans A Math Phys Eng Sci. 367(1904):4077–4095. doi:10.1098/rsta.2009.0108. 19736234

[CIT0081] Kumarasamy KK, Toleman MA, Walsh TR, Bagaria J, Butt F, Balakrishnan R, Chaudhary U, Doumith M, Giske CG, Irfan S, et al. 2010. Emergence of a new antibiotic resistance mechanism in India, Pakistan, and the UK: a molecular, biological, and epidemiological study. Lancet Infect Dis. 10(9):597–602. doi:10.1016/S1473-3099(10)70143-2. 20705517 PMC2933358

[CIT0082] Lam MMC, Wyres KL, Wick RR, Judd LM, Fostervold A, Holt KE, Lohr IH. 2019. Convergence of virulence and MDR in a single plasmid vector in MDR *Klebsiella pneumoniae* ST15. J Antimicrob Chemother. 74(5):1218–1222. doi:10.1093/jac/dkz028. 30770708 PMC6477991

[CIT0083] Larsson DGJ, Flach C-F. 2022. Antibiotic resistance in the environment. Nat Rev Microbiol. 20(5):257–269. doi:10.1038/s41579-021-00649-x. 34737424 PMC8567979

[CIT0084] Law Y, Ye L, Pan Y, Yuan Z. 2012. Nitrous oxide emissions from wastewater treatment processes. Philos Trans R Soc Lond B Biol Sci. 367(1593):1265–1277. doi:10.1098/rstb.2011.0317. 22451112 PMC3306625

[CIT0085] Li B, Qiu Y, Song Y, Lin H, Yin H. 2019. Dissecting horizontal and vertical gene transfer of antibiotic resistance plasmid in bacterial community using microfluidics. Environ Int. 131:105007. doi:10.1016/j.envint.2019.105007. 31326825

[CIT0086] Li W, Zhang WG, Zhang MS, Lei ZF, Li PF, Ma Y, Gao Y. 2022. Environmentally relevant concentrations of mercury facilitate the horizontal transfer of plasmid-mediated antibiotic resistance genes. Sci Total Environ. 852:158272. doi:10.1016/j.scitotenv.2022.158272. 36028018

[CIT0087] Li H, Dechesne A, He Z, Jensen MM, Song HL, Smets BF. 2023a. Electrochemical disinfection may increase the spread of antibiotic resistance genes by promoting conjugal plasmid transfer. Sci Total Environ. 858(Pt 1):159846. doi:10.1016/j.scitotenv.2022.159846. 36328265

[CIT0088] Li X, Xue X, Jia J, Zou X, Guan Y, Zhu L, Wang Z. 2023b. Nonsteroidal anti-inflammatory drug diclofenac accelerates the emergence of antibiotic resistance via mutagenesis. Environ Pollut. 326:121457. doi:10.1016/j.envpol.2023.121457. 36958653

[CIT0089] Liebert CA, Hall RM, Summers AO. 1999. Transposon Tn*21*, flagship of the floating genome. Microbiol Mol Biol Rev. 63(3):507–522. doi:10.1128/MMBR.63.3.507-522.1999. 10477306 PMC103744

[CIT0090] Lin H, Jiang L, Li B, Dong Y, He Y, Qiu Y. 2019. Screening and evaluation of heavy metals facilitating antibiotic resistance gene transfer in a sludge bacterial community. Sci Total Environ. 695:133862. doi:10.1016/j.scitotenv.2019.133862. 31425984

[CIT0091] Ling Z, Yin W, Shen Z, Wang Y, Shen J, Walsh TR. 2020. Epidemiology of mobile colistin resistance genes *mcr-1* to *mcr-9*. J Antimicrob Chemother. 75(11):3087–3095. doi:10.1093/jac/dkaa205. 32514524

[CIT0092] Liu J, Zhang L, Lu G, Jiang R, Yan Z, Li Y. 2021. Occurrence, toxicity and ecological risk of Bisphenol A analogues in aquatic environment - a review. Ecotoxicol Environ Saf. 208:111481. doi:10.1016/j.ecoenv.2020.111481. 33120264

[CIT0093] Liu X, Khara P, Baker ML, Christie PJ, Hu B. 2022. Structure of a type IV secretion system core complex encoded by multi-drug resistance F plasmids. Nat Commun. 13(1):379. doi:10.1038/s41467-022-28058-5. 35046412 PMC8770708

[CIT0094] Liu Y, Tong Z, Shi J, Jia Y, Yang K, Wang Z. 2020. Correlation between exogenous compounds and the horizontal transfer of plasmid-borne antibiotic resistance genes. Microorganisms. 8(8):1211. doi:10.3390/microorganisms8081211. 32784449 PMC7463591

[CIT0095] Liu Y-Y, Wang Y, Walsh TR, Yi L-X, Zhang R, Spencer J, Doi Y, Tian G, Dong B, Huang X, et al. 2016. Emergence of plasmid-mediated colistin resistance mechanism MCR-1 in animals and human beings in China: a microbiological and molecular biological study. Lancet Infect Dis. 16(2):161–168. doi:10.1016/S1473-3099(15)00424-7. 26603172

[CIT0096] Lopatkin AJ, Huang S, Smith RP, Srimani JK, Sysoeva TA, Bewick S, Karig DK, You L. 2016. Antibiotics as a selective driver for conjugation dynamics. Nat Microbiol. 1(6):16044. doi:10.1038/nmicrobiol.2016.44. 27572835 PMC5010019

[CIT0097] Low HH, Gubellini F, Rivera-Calzada A, Braun N, Connery S, Dujeancourt A, Lu F, Redzej A, Fronzes R, Orlova EV, et al. 2014. Structure of a type IV secretion system. Nature. 508(7497):550–553. doi:10.1038/nature13081. 24670658 PMC3998870

[CIT0098] Low WW, Wong JLC, Beltran LC, Seddon C, David S, Kwong H-S, Bizeau T, Wang F, Peña A, Costa TRD, et al. 2022. Mating pair stabilization mediates bacterial conjugation species specificity. Nat Microbiol. 7(7):1016–1027. doi:10.1038/s41564-022-01146-4. 35697796 PMC9246713

[CIT0099] Lu J, Ding P, Wang Y, Guo J. 2022a. Antidepressants promote the spread of extracellular antibiotic resistance genes via transformation. ISME Commun. 2(1):63. doi:10.1038/s43705-022-00147-y. 37938640 PMC9330934

[CIT0100] Lu X, Du Y, Peng K, Zhang W, Li J, Wang Z, Li R. 2022b. Coexistence of *tet*(X4), *mcr-1*, and *bla*NDM-5 in ST6775 *Escherichia coli* isolates of animal origin in China. Microbiol Spectr. 10(2):e0019622. doi:10.1128/spectrum.00196-22. 35311537 PMC9045152

[CIT0101] Lu Y, Zeng J, Wang L, Lan K, E S, Wang L, Xiao Q, Luo Q, Huang X, Huang B, et al. 2017. Antibiotics promote *Escherichia coli*-*Pseudomonas aeruginosa* conjugation through inhibiting quorum sensing. Antimicrob Agents Chemother. 61(12):1–7. doi:10.1128/AAC.01284-17. PMC5700318 28993333

[CIT0102] Macé K, Vadakkepat AK, Redzej A, Lukoyanova N, Oomen C, Braun N, Ukleja M, Lu F, Costa TRD, Orlova EV, et al. 2022. Cryo-EM structure of a type IV secretion system. Nature. 607(7917):191–196. doi:10.1038/s41586-022-04859-y. 35732732 PMC9259494

[CIT0103] Mandi TY, Molnar J, Holland IB, Beladi I. 1975. Efficient curing of an *Escherichia coli* F-prime plasmid by phenothiazines. Genet Res. 26(1):109–111. doi:10.1017/s0016672300015895. 767215

[CIT0104] Mc Mahon MA, Blair IS, Moore JE, Mc Dowell DA. 2007. The rate of horizontal transmission of antibiotic resistance plasmids is increased in food preservation-stressed bacteria. J Appl Microbiol. 103(5):1883–1888. doi:10.1111/j.1365-2672.2007.03412.x. 17953597

[CIT0105] McGovern AS, Hamlin AS, Winter G. 2019. A review of the antimicrobial side of antidepressants and its putative implications on the gut microbiome. Aust N Z J Psychiatry. 53(12):1151–1166. doi:10.1177/0004867419877954. 31558039

[CIT0106] McHugh GL, Swartz MN. 1977. Elimination of plasmids from several bacterial species by novobiocin. Antimicrob Agents Chemother. 12(3):423–426. doi:10.1128/AAC.12.3.423. 907332 PMC429929

[CIT0107] McInnes RS, McCallum GE, Lamberte LE, van Schaik W. 2020. Horizontal transfer of antibiotic resistance genes in the human gut microbiome. Curr Opin Microbiol. 53:35–43. doi:10.1016/j.mib.2020.02.002. 32143027

[CIT0108] McKenzie GJ, Harris RS, Lee PL, Rosenberg SM. 2000. The SOS response regulates adaptive mutation. Proc Natl Acad Sci U S A. 97(12):6646–6651. doi:10.1073/pnas.120161797. 10829077 PMC18688

[CIT0109] Michel-Briand Y, Uccelli V, Laporte J-M, Plesiat P. 1986. Elimination of plasmids from Enterobacteriaceae by 4-quinolone derivatives. J Antimicrob Chemother. 18(6):667–674. doi:10.1093/jac/18.6.667. 3029010

[CIT0110] Mohanalakshmi S, Bhatt S, Ashok Kumar CK. 2021. Enhanced antihyperlipidemic potential of gemfibrozil under co-administration with piperine. Curr Res Pharmacol Drug Discov. 2:100021. doi:10.1016/j.crphar.2021.100021. 34909656 PMC8663971

[CIT0111] Mohanraj RS, Mandal J. 2022. Azithromycin can induce SOS response and horizontal gene transfer of SXT element in *Vibrio cholerae*. Mol Biol Rep. 49(6):4737–4748. doi:10.1007/s11033-022-07323-2. 35286518

[CIT0112] Moller TSB, Liu G, Boysen A, Thomsen LE, Luthje FL, Mortensen S, Moller-Jensen J, Olsen JE. 2017. Treatment with cefotaxime affects expression of conjugation associated proteins and conjugation transfer frequency of an IncI1 plasmid in *Escherichia coli*. Front Microbiol. 8:2365. doi:10.3389/fmicb.2017.02365. 29238335 PMC5712592

[CIT0113] Molnar J, Mandi Y, Kiraly J. 1976. Antibacterial effect of some phenothiazine compounds and R-factor elimination by chlorpromazine. Acta Microbiol Acad Sci Hung. 23:45–54. 820163

[CIT0114] Munoz-Bellido JL, Munoz-Criado S, Garcìa-Rodrìguez JA. 2000. Antimicrobial activity of psychotropic drugs: selective serotonin reuptake inhibitors. Int J Antimicrob Agents. 14(3):177–180. doi:10.1016/s0924-8579(99)00154-5. 10773485

[CIT0115] Neil K, Allard N, Rodrigue S. 2021. Molecular mechanisms influencing bacterial conjugation in the intestinal microbiota. Front Microbiol. 12:673260. doi:10.3389/fmicb.2021.673260. 34149661 PMC8213034

[CIT0116] Nolen WA, Jansen GS, Broekman M. 1988. Measuring plasma levels of carbamazepine. A pharmacokinetic study in patients with affective disorders. Pharmacopsychiatry. 21(5):252–254. doi:10.1055/s-2007-1016965. 3227056

[CIT0117] Norman A, Hansen LH, Sorensen SJ. 2009. Conjugative plasmids: vessels of the communal gene pool. Philos Trans R Soc Lond B Biol Sci. 364(1527):2275–2289. doi:10.1098/rstb.2009.0037. 19571247 PMC2873005

[CIT0118] Ott LC, Mellata M. 2022. Models for gut-mediated horizontal gene transfer by bacterial plasmid conjugation. Front Microbiol. 13:891548. doi:10.3389/fmicb.2022.891548. 35847067 PMC9280185

[CIT0119] Ou J, Elizalde P, Guo HB, Qin H, Tobe BTD, Choy JS. 2022. TCA and SSRI antidepressants exert selection pressure for efflux-dependent antibiotic resistance mechanisms in *Escherichia coli*. mBio. 13(6):e0219122. doi:10.1128/mbio.02191-22. 36374097 PMC9765716

[CIT0120] Pal C, Asiani K, Arya S, Rensing C, Stekel DJ, Larsson DGJ, Hobman JL. 2017. Metal resistance and its association with antibiotic resistance. Adv Microb Physiol. 70:261–313. doi:10.1016/bs.ampbs.2017.02.001. 28528649

[CIT0121] Partridge SR, Kwong SM, Firth N, Jensen SO. 2018. Mobile genetic elements associated with antimicrobial resistance. Clin Microbiol Rev. 31(4):1–61. doi:10.1128/CMR.00088-17. PMC6148190 30068738

[CIT0122] Patangia DV, Anthony Ryan C, Dempsey E, Paul Ross R, Stanton C. 2022. Impact of antibiotics on the human microbiome and consequences for host health. Microbiologyopen. 11(1):e1260. doi:10.1002/mbo3.1260. 35212478 PMC8756738

[CIT0123] Penders J, Stobberingh EE, Savelkoul PH, Wolffs PF. 2013. The human microbiome as a reservoir of antimicrobial resistance. Front Microbiol. 4:87. doi:10.3389/fmicb.2013.00087. 23616784 PMC3627978

[CIT0124] Peruzzo PJ, Porta AA, Ronco AE. 2008. Levels of glyphosate in surface waters, sediments and soils associated with direct sowing soybean cultivation in north pampasic region of Argentina. Environ Pollut. 156(1):61–66. doi:10.1016/j.envpol.2008.01.015. 18308436

[CIT0125] Pilla G, Tang CM. 2018. Going around in circles: virulence plasmids in enteric pathogens. Nat Rev Microbiol. 16(8):484–495. doi:10.1038/s41579-018-0031-2. 29855597

[CIT0126] Pu Q, Fan XT, Li H, An XL, Lassen SB, Su JQ. 2021. Cadmium enhances conjugative plasmid transfer to a fresh water microbial community. Environ Pollut. 268(Pt B):115903. doi:10.1016/j.envpol.2020.115903. 33120155

[CIT0127] Qiu W, Zhan H, Hu J, Zhang T, Xu H, Wong M, Xu B, Zheng C. 2019. The occurrence, potential toxicity, and toxicity mechanism of bisphenol S, a substitute of bisphenol A: a critical review of recent progress. Ecotoxicol Environ Saf. 173:192–202. doi:10.1016/j.ecoenv.2019.01.114. 30772709

[CIT0128] Qiu Z, Yu Y, Chen Z, Jin M, Yang D, Zhao Z, Wang J, Shen Z, Wang X, Qian D, et al. 2012. Nanoalumina promotes the horizontal transfer of multiresistance genes mediated by plasmids across genera. Proc Natl Acad Sci U S A. 109(13):4944–4949. doi:10.1073/pnas.1107254109. 22411796 PMC3323979

[CIT0129] Ramsay KA, McTavish SM, Wardell SJT, Lamont IL. 2021. The effects of sub-inhibitory antibiotic concentrations on *Pseudomonas aeruginosa*: reduced susceptibility due to mutations. Front Microbiol. 12:789550. doi:10.3389/fmicb.2021.789550. 34987489 PMC8721600

[CIT0130] Ribeiro E, Ladeira C, Viegas S. 2017. Occupational exposure to bisphenol A (BPA): a reality that still needs to be unveiled. Toxics. 5(3):22. doi:10.3390/toxics5030022. 29051454 PMC5634705

[CIT0131] Ripoll-Rozada J, Garcia-Cazorla Y, Getino M, Machon C, Sanabria-Rios D, de la Cruz F, Cabezon E, Arechaga I. 2016. Type IV traffic ATPase TrwD as molecular target to inhibit bacterial conjugation. Mol Microbiol. 100(5):912–921. doi:10.1111/mmi.13359. 26915347 PMC4908816

[CIT0132] Rosas NC, Lithgow T. 2022. Targeting bacterial outer-membrane remodelling to impact antimicrobial drug resistance. Trends Microbiol. 30(6):544–552. doi:10.1016/j.tim.2021.11.002. 34872824

[CIT0133] Rozwandowicz M, Brouwer MSM, Fischer J, Wagenaar JA, Gonzalez-Zorn B, Guerra B, Mevius DJ, Hordijk J. 2018. Plasmids carrying antimicrobial resistance genes in Enterobacteriaceae. J Antimicrob Chemother. 73(5):1121–1137. doi:10.1093/jac/dkx488. 29370371

[CIT0134] Rukavishnikov G, Leonova L, Kasyanov E, Leonov V, Neznanov N, Mazo G. 2023. Antimicrobial activity of antidepressants on normal gut microbiota: results of the *in vitro* study. Front Behav Neurosci. 17:1132127. doi:10.3389/fnbeh.2023.1132127. 37035624 PMC10073483

[CIT0135] Samuels AL, Lanka E, Davies JE. 2000. Conjugative junctions in RP4-mediated mating of *Escherichia coli*. J Bacteriol. 182(10):2709–2715. doi:10.1128/JB.182.10.2709-2715.2000. 10781537 PMC101974

[CIT0136] San Millan A. 2018. Evolution of plasmid-mediated antibiotic resistance in the clinical context. Trends Microbiol. 26(12):978–985. doi:10.1016/j.tim.2018.06.007. 30049587

[CIT0137] San Millan A, Toll-Riera M, Qi Q, Betts A, Hopkinson RJ, McCullagh J, MacLean RC. 2018. Integrative analysis of fitness and metabolic effects of plasmids in *Pseudomonas aeruginosa* PAO1. ISME J. 12(12):3014–3024. doi:10.1038/s41396-018-0224-8. 30097663 PMC6246594

[CIT0138] Sanchez-Cid C, Guironnet A, Keuschnig C, Wiest L, Vulliet E, Vogel TM. 2022. Gentamicin at sub-inhibitory concentrations selects for antibiotic resistance in the environment. ISME Commun. 2(1):29. doi:10.1038/s43705-022-00101-y. 37938295 PMC9723587

[CIT0139] Sanderson KE, Janzer J, Head J. 1981. Influence of lipopolysaccharide and protein in the cell envelope on recipient capacity in conjugation of *Salmonella* typhimurium. J Bacteriol. 148(1):283–293. doi:10.1128/jb.148.1.283-293.1981. 7026536 PMC216191

[CIT0140] Seitz P, Blokesch M. 2013. Cues and regulatory pathways involved in natural competence and transformation in pathogenic and environmental Gram-negative bacteria. FEMS Microbiol Rev. 37(3):336–363. doi:10.1111/j.1574-6976.2012.00353.x. 22928673

[CIT0141] Sengupta M, Austin S. 2011. Prevalence and significance of plasmid maintenance functions in the virulence plasmids of pathogenic bacteria. Infect Immun. 79(7):2502–2509. doi:10.1128/IAI.00127-11. 21555398 PMC3191983

[CIT0142] Shah PN, Marshall-Batty KR, Smolen JA, Tagaev JA, Chen Q, Rodesney CA, Le HH, Gordon VD, Greenberg DE, Cannon CL. 2018. Antimicrobial activity of ibuprofen against cystic fibrosis-associated gram-negative pathogens. Antimicrob Agents Chemother. 62(3):1–22. doi:10.1128/AAC.01574-17. PMC5826130 29311081

[CIT0143] Shen Z, Tang CM, Liu G-Y. 2022. Towards a better understanding of antimicrobial resistance dissemination: what can be learnt from studying model conjugative plasmids? Mil Med Res. 9(1):3. doi:10.1186/s40779-021-00362-z. 35012680 PMC8744291

[CIT0144] Shun-Mei E, Zeng JM, Yuan H, Lu Y, Cai RX, Chen C. 2018. Sub-inhibitory concentrations of fluoroquinolones increase conjugation frequency. Microb Pathog. 114:57–62. doi:10.1016/j.micpath.2017.11.036. 29174700

[CIT0145] Smillie C, Garcillan-Barcia MP, Francia MV, Rocha EP, de la Cruz F. 2010. Mobility of plasmids. Microbiol Mol Biol Rev. 74(3):434–452. doi:10.1128/MMBR.00020-10. 20805406 PMC2937521

[CIT0146] Sommer MOA, Dantas G, Church GM. 2009. Functional characterization of the antibiotic resistance reservoir in the human microflora. Science. 325(5944):1128–1131. doi:10.1126/science.1176950. 19713526 PMC4720503

[CIT0147] Sørensen SJ, Bailey M, Hansen LH, Kroer N, Wuertz S. 2005. Studying plasmid horizontal transfer in situ: a critical review. Nat Rev Microbiol. 3(9):700–710. doi:10.1038/nrmicro1232. 16138098

[CIT0148] Stage TB, Bergmann TK, Kroetz DL. 2018. Clinical pharmacokinetics of paclitaxel monotherapy: an updated literature review. Clin Pharmacokinet. 57(1):7–19. doi:10.1007/s40262-017-0563-z. 28612269 PMC8572663

[CIT0149] Stalder T, Top E. 2016. Plasmid transfer in biofilms: a perspective on limitations and opportunities. NPJ Biofilms Microbiomes. 2:16022–16022. doi:10.1038/npjbiofilms.2016.22. 28480050 PMC5416938

[CIT0150] Sun J, Chen C, Cui C-Y, Zhang Y, Liu X, Cui Z-H, Ma X-Y, Feng Y, Fang L-X, Lian X-L, et al. 2019. Plasmid-encoded *tet*(X) genes that confer high-level tigecycline resistance in *Escherichia coli*. Nat Microbiol. 4(9):1457–1464. doi:10.1038/s41564-019-0496-4. 31235960 PMC6707864

[CIT0151] Suzuki S, Kimura M, Agusa T, Rahman HM. 2012. Vanadium accelerates horizontal transfer of *tet*(M) gene from marine *Photobacterium* to *Escherichia coli*. FEMS Microbiol Lett. 336(1):52–56. doi:10.1111/j.1574-6968.2012.02653.x. 22889204

[CIT0152] Tang H, Liu Z, Hu B, Zhu L. 2022. Effects of iron mineral adhesion on bacterial conjugation: interfering the transmission of antibiotic resistance genes through an interfacial process. J Hazard Mater. 435:128889. doi:10.1016/j.jhazmat.2022.128889. 35472548

[CIT0153] Thomas CM, Nielsen KM. 2005. Mechanisms of, and barriers to, horizontal gene transfer between bacteria. Nat Rev Microbiol. 3(9):711–721. doi:10.1038/nrmicro1234. 16138099

[CIT0154] Thomson VJ, Jovanovic OS, Pohlman RF, Chang CH, Figurski DH. 1993. Structure, function, and regulation of the *kilB* locus of promiscuous plasmid RK2. J Bacteriol. 175(8):2423–2435. doi:10.1128/jb.175.8.2423-2435.1993. 8468300 PMC204532

[CIT0155] Van Bruggen AHC, He MM, Shin K, Mai V, Jeong KC, Finckh MR, Morris JG. Jr 2018. Environmental and health effects of the herbicide glyphosate. Sci Total Environ. 616-617:255–268. doi:10.1016/j.scitotenv.2017.10.309. 29117584

[CIT0156] Vats P, Kaur UJ, Rishi P. 2022. Heavy metal‐induced selection and proliferation of antibiotic resistance: A review. J Appl Microbiol. 132(6):4058–4076. doi:10.1111/jam.15492. 35170159

[CIT0157] Vich Vila A, Collij V, Sanna S, Sinha T, Imhann F, Bourgonje AR, Mujagic Z, Jonkers DMAE, Masclee AAM, Fu J, et al. 2020. Impact of commonly used drugs on the composition and metabolic function of the gut microbiota. Nat Commun. 11(1):362. doi:10.1038/s41467-019-14177-z. 31953381 PMC6969170

[CIT0158] Virolle C, Goldlust K, Djermoun S, Bigot S, Lesterlin C. 2020. Plasmid transfer by conjugation in gram-negative bacteria: from the cellular to the community level. Genes (Basel). 11(11):1239. doi:10.3390/genes11111239. 33105635 PMC7690428

[CIT0159] Waksman G. 2019. From conjugation to T4S systems in Gram-negative bacteria: a mechanistic biology perspective. EMBO Rep. 20:1–16. 10.15252/embr.201847012 PMC6362355 30602585

[CIT0160] Walsh J, Griffin BT, Clarke G, Hyland NP. 2018. Drug-gut microbiota interactions: implications for neuropharmacology. Br J Pharmacol. 175(24):4415–4429. doi:10.1111/bph.14366. 29782640 PMC6255959

[CIT0161] Wang C, Feng Y, Liu L, Wei L, Kang M, Zong Z. 2020a. Identification of novel mobile colistin resistance gene *mcr-10*. Emerg Microbes Infect. 9(1):508–516. doi:10.1080/22221751.2020.1732231. 32116151 PMC7067168

[CIT0162] Wang Q, Mao D, Mu Q, Luo Y. 2015. Enhanced horizontal transfer of antibiotic resistance genes in freshwater microcosms induced by an ionic liquid. PLoS One. 10(5):e0126784. doi:10.1371/journal.pone.0126784. 25951456 PMC4423773

[CIT0163] Wang Q, Liu L, Hou Z, Wang L, Ma D, Yang G, Guo S, Luo J, Qi L, Luo Y. 2020b. Heavy metal copper accelerates the conjugative transfer of antibiotic resistance genes in freshwater microcosms. Sci Total Environ. 717:137055. doi:10.1016/j.scitotenv.2020.137055. 32065888

[CIT0164] Wang X, Chen Z, Mu Q, Wu X, Zhang J, Mao D, Luo Y, Alvarez PJJ. 2020c. Ionic liquid enriches the antibiotic resistome, especially efflux pump genes, before significantly affecting microbial community structure. Environ Sci Technol. 54(7):4305–4315. doi:10.1021/acs.est.9b04116. 31944684

[CIT0165] Wang Y, Lu J, Mao L, Li J, Yuan Z, Bond PL, Guo J. 2019. Antiepileptic drug carbamazepine promotes horizontal transfer of plasmid-borne multi-antibiotic resistance genes within and across bacterial genera. ISME J. 13(2):509–522. doi:10.1038/s41396-018-0275-x. 30291330 PMC6331567

[CIT0166] Wang Y, Lu J, Zhang S, Li J, Mao L, Yuan Z, Bond PL, Guo J. 2021. Non-antibiotic pharmaceuticals promote the transmission of multidrug resistance plasmids through intra- and intergenera conjugation. ISME J. 15(9):2493–2508. doi:10.1038/s41396-021-00945-7. 33692486 PMC8397710

[CIT0167] Wang Y, Yu Z, Ding P, Lu J, Klümper U, Murray AK, Gaze WH, Guo J. 2022. Non-antibiotic pharmaceuticals promote conjugative plasmid transfer at a community-wide level. Microbiome. 10(1):124. doi:10.1186/s40168-022-01314-y. 35953866 PMC9373378

[CIT0168] Wang Y, Lu J, Engelstädter J, Zhang S, Ding P, Mao L, Yuan Z, Bond PL, Guo J. 2020d. Non-antibiotic pharmaceuticals enhance the transmission of exogenous antibiotic resistance genes through bacterial transformation. ISME J. 14(8):2179–2196. doi:10.1038/s41396-020-0679-2. 32424247 PMC7367833

[CIT0169] Wang Y, Yu Z, Ding P, Lu J, Mao L, Ngiam L, Yuan Z, Engelstadter J, Schembri MA, Guo J. 2023. Antidepressants can induce mutation and enhance persistence toward multiple antibiotics. Proc Natl Acad Sci U S A. 120(5):e2208344120. doi:10.1073/pnas.2208344120. 36689653 PMC9945972

[CIT0170] Wang Y-F, Qiao M, Zhu D, Zhu Y-G. 2020e. Antibiotic resistance in the collembolan gut microbiome accelerated by the nonantibiotic drug carbamazepine. Environ Sci Technol. 54(17):10754–10762. doi:10.1021/acs.est.0c03075. 32816468

[CIT0171] Weersma RK, Zhernakova A, Fu J. 2020. Interaction between drugs and the gut microbiome. Gut. 69(8):1510–1519. doi:10.1136/gutjnl-2019-320204. 32409589 PMC7398478

[CIT0172] Weise K, Winter L, Fischer E, Kneis D, de la Cruz Barron M, Kunze S, Berendonk TU, Jungmann D, Klumper U. 2022. Multiwalled carbon nanotubes promote bacterial conjugative plasmid transfer. Microbiol Spectr. 10(2):e0041022. doi:10.1128/spectrum.00410-22. 35384690 PMC9045119

[CIT0173] Weisser J, Wiedemann B. 1985. Elimination of plasmids by new 4-quinolones. Antimicrob Agents Chemother. 28(5):700–702. doi:10.1128/AAC.28.5.700. 3911881 PMC176363

[CIT0174] Winter M, Buckling A, Harms K, Johnsen PJ, Vos M. 2021. Antimicrobial resistance acquisition via natural transformation: context is everything. Curr Opin Microbiol. 64:133–138. doi:10.1016/j.mib.2021.09.009. 34710742

[CIT0175] Xiao X, Zeng F, Li R, Liu Y, Wang Z. 2022. subinhibitory concentration of colistin promotes the conjugation frequencies of *mcr*-1- and *bla*NDM-5-positive plasmids. Microbiol Spectr. 10(2):e02160-02121. doi:10.1128/spectrum.02160-21. PMC9045390 35230128

[CIT0176] Xie M, Yang X, Xu Q, Ye L, Chen K, Zheng Z, Dong N, Sun Q, Shu L, Gu D, et al. 2021. Clinical evolution of ST11 carbapenem resistant and hypervirulent *Klebsiella pneumoniae*. Commun Biol. 4(1):650. doi:10.1038/s42003-021-02148-4. 34075192 PMC8169677

[CIT0177] Yang B, Wang Z, Jia Y, Fang D, Li R, Liu Y. 2022. Paclitaxel and its derivative facilitate the transmission of plasmid-mediated antibiotic resistance genes through conjugative transfer. Sci Total Environ. 810:152245. doi:10.1016/j.scitotenv.2021.152245. 34896514

[CIT0178] Yang QE, Agouri SR, Tyrrell JM, Walsh TR. 2018. Heavy metal resistance genes are associated with *bla*(NDM-1)- and *bla*(CTX-M-15)-carrying Enterobacteriaceae. Antimicrob Agents Chemother. 62(5):1–7. doi:10.1128/AAC.02642-17. PMC5923091 29507071

[CIT0179] Yaseen DA, Scholz M. 2019. Textile dye wastewater characteristics and constituents of synthetic effluents: a critical review. Int J Environ Sci Technol. 16(2):1193–1226. doi:10.1007/s13762-018-2130-z.

[CIT0180] Yu Z, Henderson IR, Guo J. 2023. Non-caloric artificial sweeteners modulate conjugative transfer of multi-drug resistance plasmid in the gut microbiota. Gut Microbes. 15(1):2157698. doi:10.1080/19490976.2022.2157698. 36524841 PMC9762752

[CIT0181] Yu Z, Wang Y, Henderson IR, Guo J. 2022. Artificial sweeteners stimulate horizontal transfer of extracellular antibiotic resistance genes through natural transformation. ISME J. 16(2):543–554. doi:10.1038/s41396-021-01095-6. 34465899 PMC8776823

[CIT0182] Yu Z, Wang Y, Lu J, Bond PL, Guo J. 2021. Nonnutritive sweeteners can promote the dissemination of antibiotic resistance through conjugative gene transfer. ISME J. 15(7):2117–2130. doi:10.1038/s41396-021-00909-x. 33589766 PMC8245538

[CIT0183] Zhang H, Liu J, Wang L, Zhai Z. 2021. Glyphosate escalates horizontal transfer of conjugative plasmid harboring antibiotic resistance genes. Bioengineered. 12(1):63–69. doi:10.1080/21655979.2020.1862995. 33345705 PMC8806241

[CIT0184] Zhang PY, Xu PP, Xia ZJ, Wang J, Xiong J, Li YZ. 2013. Combined treatment with the antibiotics kanamycin and streptomycin promotes the conjugation of *Escherichia coli*. FEMS Microbiol Lett. 348(2):149–156. doi:10.1111/1574-6968.12282. 24111668

[CIT0185] Zhang S, Wang Y, Song H, Lu J, Yuan Z, Guo J. 2019. Copper nanoparticles and copper ions promote horizontal transfer of plasmid-mediated multi-antibiotic resistance genes across bacterial genera. Environ Int. 129:478–487. doi:10.1016/j.envint.2019.05.054. 31158594

[CIT0186] Zhang Y, Geissen S-U, Gal C. 2008. Carbamazepine and diclofenac: removal in wastewater treatment plants and occurrence in water bodies. Chemosphere. 73(8):1151–1161. doi:10.1016/j.chemosphere.2008.07.086. 18793791

[CIT0187] Zhang Y, Gu AZ, Cen T, Li X, He M, Li D, Chen J. 2018. Sub-inhibitory concentrations of heavy metals facilitate the horizontal transfer of plasmid-mediated antibiotic resistance genes in water environment. Environ Pollut. 237:74–82. doi:10.1016/j.envpol.2018.01.032. 29477117

[CIT0188] Zheng B, Dong H, Xu H, Lv J, Zhang J, Jiang X, Du Y, Xiao Y, Li L. 2016. Coexistence of MCR-1 and NDM-1 in clinical *Escherichia coli* isolates. Clin Infect Dis. 63(10):1393–1395. doi:10.1093/cid/ciw553. 27506685

[CIT0189] Zhou Y, Ai W, Cao Y, Guo Y, Wu X, Wang B, Rao L, Xu Y, Zhao H, Wang X, et al. 2021. The Co-occurrence of NDM-5, MCR-1, and FosA3-Encoding Plasmids Contributed to the Generation of Extensively Drug-Resistant *Klebsiella pneumoniae*. Front Microbiol. 12:811263. doi:10.3389/fmicb.2021.811263. 35046925 PMC8762306

