# Supplementary material for: Non-antibiotic compounds associated with humans and the environment can promote horizontal transfer of antimicrobial resistance genes
Source: Crit Rev Microbiol. 2023 Jul 18;50(6):993–1010. doi: 10.1080/1040841X.2023.2233603 (PMC11523920; doi:10.1080/1040841X.2023.2233603)
Supplement: Supplemental Material [file IMBY_A_2233603_SM6299.docx]

**Table S1.** List of non-antibiotic pharmaceuticals and food additives that have been reported to promote horizontal transfer of AMR genes in bacteria.

| **Compound (s)** | **Species^a^** | **Plasmid and characteristics** | **Key findings** | **Reference** |
| --- | --- | --- | --- | --- |
| Antidepressants | *E. coli →* *P. putida* | RP4 (IncP⍺ plasmid)  Contains *aphA, bla*_TEM_*,* and *tet*(A) genes | 100 µg/mL bupropion: >8-fold increase in CF  10 µg/mL bupropion and duloxetine: >3-fold increase in CF  10 µg/mL sertraline: >6-fold increase in CF | (1) |
|  |  | pMS6198A (IncA/C plasmid)  Contains *ble*_MBL_, *bla*_NDM-1_, *sul1*, and *aacA4* genes | 5 µg/mL bupropion: ~2-fold increase in CF  10 µg/mL fluoxetine and duloxetine: >2-fold increase in CF  100 µg/mL escitalopram: >2-fold increase in CF  1 µg/mL sertraline: >4-fold increase in CF |  |
|  | *A. baylyi* | pWH1266  Contains *bla*_TEM-1_ and *tet*(A) genes | 1 and 10 µg/mL duloxetine: 2.3-fold increase in TR  1 µg/mL fluoxetine: >1.5-fold increase in TR  0.1 µg/mL sertraline: >1.5-fold increase in TR | (2) |
|  | *E. coli → E. coli* | pKJK5*gfp* (IncP-1 plasmid) Contains *aadA11b*, *dfrA1*, *sulA*, *tet*(A) and *gfp* genes. | 10 µg/mL sertraline: 4.6-fold increase in CF within 5 minutes | (3) |
| NSAIDs | *A. baylyi* | pWH1266 | 50 µg/mL diclofenac: 2.3-fold increase in TF  50 µg/mL ibuprofen: 1.9-fold increase in TF  50 µg/mL naproxen: 2.1-fold increase in TF | (4) |
|  | *E. coli →* *E. coli* | pMS6198A | 0.5 µg/mL diclofenac, ibuprofen, and naproxen: >2-fold increase in CF | (5) |
|  | *E. coli →* *P. putida* | RP4 | 0.005 µg/mL ibuprofen: 2.8-fold increase in CF  0.05 µg/mL naproxen: >4-fold increase in CF  50  µg/mL ibuprofen: 7.3-fold increase in CF | (5) |
|  | *P. putida →* activated sludge community | RP4 | 5 µg/mL naproxen: >2-fold increase in CF | (6) |
| Paracetamol/  Acetaminophen | *E. coli →* *E. coli* | RP4-7 (IncP⍺ plasmid)  Contains *bla*_TEM_ and *catA* genes | 50 µg/mL: >2-fold increase in CF | (7) |
|  |  | IncX4 and IncI2 plasmids carrying *mcr-1* | 50 µg/mL: >2-fold increase in CF |  |
|  |  | IncFII plasmids carrying *tet*(X4) | 50 µg/mL: ~1.5-fold increase in CF |  |
|  | *E. coli → P*seudomonas sp. HLS-6 | RP4 | 0.04 µg/mL: >4-fold increase in CF | (8) |
| Lipid-lowering drug (gemfibrozil) | *A. baylyi* | pWH1266 | 0.005 µg/mL: >2-fold increase in TF | (4) |
|  | *E. coli →* *E. coli* | pMS6198A | 0.005 µg/mL: >2-fold increase in CF | (5) |
|  | *E. coli →* *P. putida* | RP4 | 0.005 µg/mL: >4-fold increase in CF  0.05 µg/mL: >7-fold increase in CF | (5) |
|  | *P. putida →* activated sludge community | RP4 | 5 µg/mL: >2-fold increase in CF | (6) |
| β-blocker (propranolol) | *A. baylyi* | pWH1266 | 0.05 µg/mL: >2-fold increase in TF | (4) |
|  | *E. coli →* *E. coli* | pMS6198A | 0.05 µg/mL: >2-fold increase in CF | (5) |
|  | *E. coli →* *P. putida* | RP4 | 0.005 µg/mL: >1-fold increase in CF | (5) |
|  | *P. putida →* activated sludge community | RP4 | 0.5 µg/mL: >2-fold increase in CF | (6) |
| Anti-epileptic drug (carbamazepine) | *E. coli →* *E. coli*  *E. coli →* *P. putida* | RP4 | 0.05 µg/mL: >4-fold increase in CF | (9) |
|  | *P. putida → E. coli* |  | 0.05 µg/mL: >2-fold increase in CF |  |
|  | *P. putida →* activated sludge community | RP4 | 5 µg/mL: >2-fold increase in CF | (6) |
| Anticancer drug (paclitaxel) | *E. coli →* *E. coli* | RP4-7 | 1 µg/mL: >1.5-fold increase in CF | (10) |
|  |  | IncFI/IncFII plasmids carrying *tet*(X4) | 0.1 µg/mL: >2-fold increase in CF |  |
|  |  | IncI2/IncX4 plasmids carrying *mcr-1* | 0.1 µg/mL: >1.5-fold increase in CF |  |
|  | *K. pneumoniae →* *E. coli* | IncX3 plasmids carrying *bla*_NDM-1_ | 0.1 µg/mL: >1.5-fold increase in CF |  |
| Artificial sweeteners | *A. baylyi* | pWH1266 | 30 µg/mL aspartame: >1.5-fold increase in TF  3 µg/mL saccharin: >2-fold increase in TF  3 µg/mL acesulfame K: >1.5-fold increase in TF  0.3 µg/mL sucralose: >2-fold increase in TF | (11) |
|  | *B. subtilis* |  | 30 µg/mL aspartame, saccharin: >2-fold increase in TF  30 µg/mL acesulfame K, sucralose: >1.5-fold increase in TF |  |
|  | Mice faecal bacteria | pKJK5*gfp* | 30 µg/mL aspartame and sucralose: >2-fold increase in TF  30 µg/mL acesulfame K and saccharin: >1.5-fold increase in TF |  |
|  | *E. coli →* *E. coli* | RP4 | 3 µg/mL aspartame, acesulfame K, saccharin, and sucralose: >2-fold increase in CF | (12) |
|  |  | pMS6198A | 3 µg/mL acesulfame K, aspartame, saccharin, and sucralose: ≥2-fold increase in CF |  |
|  | *E. coli →* *P. putida* | RP4 | 3 µg/mL acesulfame K and sucralose: >2-fold increase in CF | (12) |
|  | *P. putida →* *E. coli* |  | 3 µg/mL aspartame and sucralose: >2-fold increase in CF |  |
|  | *E. coli →* Mice faecal bacteria | pKJK5*gfp* | 300 µg/mL saccharin: 3.9-fold increase in CF  300 µg/mL sucralose: 5.3-fold increase in CF | (13) |
|  | *E. coli → K. pneumoniae* | RP4 | 300 µg/mL aspartame: 13.1-fold increase in CF  300 µg/mL acesulfame K: 11.5-fold increase in CF  300 µg/mL sucralose: 6.7-fold increase in CF |  |
| Food preservatives | *E. coli →* *E. coli* | R386 (F1 plasmid)  Contains *tet*(A) | 4% sodium chloride: increase in CF from 2.1×10^−8^ to 1.2×10^−5^ | (14) |
|  |  | TP307 (IncI1 plasmid)  Contains apramycin, gentamycin, tobramycin, and streptomycin resistance genes | 4% sodium chloride: increase in CF from 6.8×10^−12^ to 1.2×10^−5^ |  |
|  |  | pCM184-Cm  Contains *bla*_TEM_*, catA*, and *tet*(A) genes | 5 µg/mL sodium nitrite: >2-fold increase in CF  100 µg/mL sodium benzoate: >6-fold increase in CF  10 µg/mL triclocarban: >4-fold increase in CF | (15) |
|  | *E. coli →* *S. enterica* | TP307 | 4% sodium chloride: increase in CF from 4.5×10^−12^ to 1.4×10^−7^ | (14) |

^a^*A → B* wherein *A* is the donor species and *B* is the recipient species. CF, conjugation frequency (number of transconjugants/number of recipients); NSAIDs, non-steroidal anti-inflammatory drugs; TC, transconjugant; TF, transformation frequency (number of transformants/total cell number); TR, transformation ratio (number of transformants/total number of recipients)

**Table S2.** List of environmental pollutants that have been reported to promote horizontal transfer of AMR genes in bacteria.

| **Compound (s)** | **Species^a^** | **Plasmid and characteristics** | **Key findings** | **Reference** |
| --- | --- | --- | --- | --- |
| Herbicide  (glyphosate) | *E. coli →* *E. coli* | AMR plasmid | 0.1 µg/mL: 2.26-fold increase in CF | (16) |
| Disinfection by-products | *E. coli →* *E. coli* | RP4 (IncP⍺ plasmid)  Contains *aphA, bla*_TEM_*,* and tet(A) genes | 10 µg/mL dichloroacetonitrile: ~6-fold increase in CF  25 µg/mL trichloromethane: ~5.5-fold increase in CF | (17) |
| Water treatment by-product (released nitric oxide from sodium nitroprusside) | *E. coli →* *E. coli* | RP4 | 0.031 µM: >2-fold increase in CF  0.65 µM: >9-fold increase in CF | (18) |
|  | *E. coli →* *S. enterica* |  | 0.21 µM: >3-fold increase in CF  0.65 µM: >9-fold increase in CF |  |
|  | *E. coli →* indigenous wastewater microbiota |  | 0.65 µM: 396% increase in absolute number of TC colonies |  |
| Plastic manufacturing precursors (bisphenols) | *E. coli →* *S. enterica* | RP4 | 0.1 µg/L bisphenol S: >2-fold increase in CF  10 µg/L bisphenol AF: >2-fold increase in CF | (19) |
|  | *E. coli →* *E. coli* |  | 0.1 µg/L bisphenol AF, bisphenol S: >2-fold increase in CF |  |
| Dyeing wastewater compounds | *E. coli →* *E. coli* | RP4 | 0.8 µg/L *o*-xylene: 219-fold increase in CF  1 µg/L ethylbenzene: 102-fold increase in CF | (20) |
| Metallic nanomaterials | *E. coli →* *P. putida* | RP4 | 0.1 µg/L silver nanoparticles: 1.8-fold increase in CF  5 µM nano-copper oxide: >2-fold increase in CF | (21)  (22) |
|  | *E. coli → E. coli* |  | 1 mM nano-titanium oxide: >150-fold increase in number of TCs  5 mM nanoalumina: >200-fold increase in CF | (23)  (24) |
|  | *E. coli →* *S. enterica* |  | 5 mM nanoalumina: >100-fold increase in CF |  |
|  | *E. coli → Enterococci* |  | 5 mM nanoalumina: >50-fold increase in CF |  |
|  | *Enterococci → Enterococci* |  | 5 mM nanoalumina: >100-fold increase in CF |  |
|  | *E. coli → E. coli* | RK2 (IncP plasmid)  Contains *aphA, bla*_TEM_ and tet(A) genes | 5 mM: nanoalumina >170-fold increase in CF |  |
|  | *Enterococci → Enterococci* | pCF10 (MOB_P7_ plasmid)  Contains tet(A) | 5 mM nanoalumina: >120-fold increase in CF |  |
| Ionic liquids | *E. coli →* freshwater bacterial community (*Acinetobacter* and *Salmonella* spp. detected) | RP4 | 1 mg/mL BMIM-PF_6_: ~60-fold increase CF | (25) |
|  |  | RK2*gfp* | 1 mg/mL BMIM-PF_6_: 60.3-fold increase in CF | (26) |
|  | *E. coli →* *S. enterica* | RP4 | 1 mg/mL BMIM-BF_4_: 5.9-fold increase in CF  0.01 mg/mL HMIM-BF_4_: 2.2-fold increase in CF  0.1 µg/mL OMIM-BF_4_: 1.7-fold increase in CF | (27) |
| Multiwalled carbon nanotubes | *P. putida →* *P. putida* | pKJK5 (IncP-1 plasmid) Contains *aadA11b*, *dfrA1*, *sulA*, and tet(A) genes. | 10 µg/mL: 2-fold increase in CF | (28) |
|  | *E. coli →* *P. putida* | pB10 (IncP-1β plasmid)  Contains *bla_O_*_XA-2_, *sul1* and tet(A) genes | 10 µg/mL: >5-fold increase in CF |  |
| Microplastics | *E. coli →* *Pseudomonas* sp. | pJKJ5*gfp* | 50 microplastic particles/100 mL microcosm: increase in CF from 2.5 ± 2.9×10^−6^ in control group to 8.2 ± 9.0×10^−3^ in treated group | (29) |
| Heavy metals | P. damselae *→* *E. coli* | pAQU1 (IncA/C-like plasmid)  Contains *bla*_CARB-9_-like, *floR*, *mph*(A)-like, *mef*(A)-like, *sul2*, *tet*(M), and *tet*(B) genes | 500 µM vanadium (III): ~2-fold increase CF | (30) |
|  | *E. coli →* *E. coli* | RP4 | 0.1 µg/mL mercury (II): 5.3-fold increase in CF | (31) |
|  |  | pCM184-Cm  Contains *bla*_TEM_*, catA*, and tet(A) genes | 0.01 µg/mL copper (II) and silver (I): >2-fold increase in CF  0.1 µg/mL chromium (VI): >2-fold increase in CF | (32) |
|  |  | pRK2013 (ColE1 replicon)  Contains *aphA* gene | 50 µg/mL goethite (iron mineral): ~3-fold increase in CF  100 µg/mL ferrihydrite (iron mineral): >4-fold increase in CF  100 µg/mL hematite (iron mineral): >3-fold increase in CF | (33) |
|  | *E. coli →* sludge bacterial community | pKJK5 | 1.36 µg/mL mercury (II): >2-fold increase in CF  165.6 µg/mL lead (II): 1.25-fold increase in CF  18.2 µg/mL arsenic (V): 1.35-fold increase in CF | (34) |
|  | *E. coli →* *P. putida* | RP4 | 0.25 µg/mL copper (II): >4-fold increase in CF  1 µg/mL silver (I): 2.4-fold increase in CF | (22)  (21) |
|  | *E. coli →* freshwater bacterial community |  | 5 µg/L copper (II): 16-fold increase in CF | (35) |
|  | *P. putida →* freshwater bacterial community |  | 100 µg/mL cadmium (II): 10-fold increase in CF | (36) |
|  | *P. aeruginosa → P. aeruginosa* | *bla*_TEM_ and *bla*_SHV_ carrying AMR plasmid | 5 µg/mL arsenic: ~5-fold increase in TC frequency | (37) |
|  | *P. aeruginosa → A. baumannii* | *bla*_TEM_ and *bla*_OXA_ carrying AMR plasmid | 5 µg/mL arsenic: ~4-fold increase in TC frequency |  |
|  | *E. coli → K. pneumoniae*  *E. coli → E. coli* | *bla*_TEM_ and *bla*_NDM-1_ carrying AMR plasmid | 5 µg/mL arsenic: ~4-fold increase in TC frequency |  |

^a^*A → B* wherein *A* is the donor species and *B* is the recipient species.

CF, conjugation frequency (number of transconjugants/number of recipients); TC, transconjugant

**Table S3.** Potential level of risk posed by non-antibiotic pharmaceuticals in disseminating antimicrobial resistance genes by horizonal gene transfer.

| **Compound (s)** | **Potential level of risk^a^** | **Evidence** | **References** |
| --- | --- | --- | --- |
| Antidepressants | High (clinical) | Clinically relevant concentrations (1–100 µg/mL), escitalopram, fluoxetine, and sertraline increased the conjugative transfer of several plasmids carrying AMR genes. | (1, 2, 38) |
|  |  | Simulated daily dosage of bupropion, duloxetine, and sertraline at 10 µg/mL induced chromosomal mutations leading to resistance and persistence in the presence of multiple antibiotics in *E. coli*. | (3) |
|  |  | Clinically relevant concentration of fluoxetine (~50 µg/mL) selects for *E. coli* mutants with increased efflux pump activity, resulting in multidrug resistance. | (39) |
|  |  | Sertraline and fluoxetine exhibit antibacterial activity against some gut microbiota and pathogenic bacteria at clinically relevant concentrations. | (38, 40, 41) |
| Diclofenac | High (clinical and environmental) | Increased conjugative transfer of several plasmids carrying AMR genes at 0.5 µg/mL, which is below the reported maximum serum concentrations (0.64–0.73 µg/mL). | (5, 42) |
|  |  | Environmentally relevant concentrations (0.01 µg/mL) induced antibiotic resistance in *E. coli* by oxidative stress and SOS response. | (43) |
|  |  | Antibacterial activity against several different Gram-negative and Gram-positive bacteria at 50-200 µg/mL. | (44, 45) |
| Naproxen | High (clinical)  Low (environmental) | Increased conjugative transfer of several plasmids carrying AMR genes at 50 µg/mL, which is below the maximum serum concentration of naproxen (94–97 µg/mL). Also increased the transfer of a plasmid carrying AMR genes to an activated sludge microbial community at 5 µg/mL, but this is significantly higher than the reported environmental concentrations. | (4, 5, 46) |
| Ibuprofen | High (clinical) | Increased conjugative transfer of plasmids carrying AMR genes at 0.005–50 µg/mL. These concentrations are within the average maximum serum concentration of ibuprofen (20 µg/mL) and the reported highest environmental concentration of ibuprofen (0.01 µg/mL). | (4, 5, 47, 48) |
|  |  | Induced phenotypic antibiotic resistance in *E. coli* at 1000–2000 µg/mL, which is significantly higher than clinically relevant concentrations. | (49) |
|  |  | Has antibacterial and anti-biofilm activity against cystic fibrosis-associated pathogens. | (50) |
| Paracetamol | Low (clinical and environmental) | Increased conjugative transfer of clinical AMR plasmids at 50 µg/mL. However, this is significantly higher than the reported maximum serum concentration (10–20 µg/mL) and the highest environmental concentrations (0.08 µg/mL). | (7, 48, 51) |
| Lipid lowering drug (gemfibrozil) | High (clinical)  Low (environmental) | Increased conjugative transfer of several different plasmids carrying AMR genes at 0.005–50 µg/mL, which are within the maximum serum concentration range (30–62 µg/mL). Also increased the transfer of a plasmid carrying AMR genes to an activated sludge microbial community at 5 µg/mL, but this is significantly higher than the reported environmental concentrations (0.01–0.064 µg/mL). | (4, 5, 52, 53, 54) |
| β-blocker (propranolol) | High (clinical)  Low (environmental) | Increased conjugative transfer of several plasmids carrying AMR genes between 0.005–0.5 µg/mL, which are within the maximum serum concentration range (0.135–0.355 µg/mL). Also increased the transfer of a plasmid carrying AMR genes to an activated sludge microbial community at 0.5 µg/mL, but this is significantly higher than the reported environmental concentrations (0.7–89 ng/L). | (4, 5, 55) |
| Anti-epileptic drug (carbamazepine) | High (clinical)  Moderate (environmental) | Increased conjugative transfer of several plasmids carrying AMR genes at 0.5, 5, and 50 µg/mL. This is within the maximum serum concentration (4–12 µg/mL). Increased the transfer of a plasmid carrying AMR genes to an activated sludge microbial community at 0.5 µg/mL, but this is significantly higher than the reported environmental concentrations (0.0005–0.017 µg/mL). | (9, 56, 57) |
|  |  | Oral exposure at environmentally relevant concentrations enhanced the abundance and diversity of antibiotic resistance genes in the gut of *Folsomia candida*, a model soil hexapod. | (58) |
| Anticancer drug (paclitaxel) | High (clinical)  Low (environmental) | Increased conjugative transfer of several different clinical AMR plasmid  at 0.1 µg/mL, which is below the reported maximum serum  concentrations (5.35–15.46 µg/mL) and significantly higher than the  reported environmental concentrations (0.003 µg/L). | (10, 59, 60) |
| Artificial sweeteners | High (domestic) | At gut relevant concentrations (3–300 mg/L), saccharin, sucralose, aspartame, and acesulfame potassium increased reactive oxygen species production and promoted plasmid-mediated conjugative transfer in mouse gut microbiota. | (11, 12, 13, 61) |
|  |  | At high concentrations (26.6 mg/mL), acesulfame potassium inhibits growth of multidrug resistant bacteria and potentiates activity of antibiotics. | (62) |
| Food preservatives | Insufficient data | At food concentration levels, sodium nitrite, sodium benzoate, and triclocarban increased conjugative transfer of a cloning vector. Insufficient data on clinically/environmentally relevant plasmids. | (15) |

^a^**Low risk:** increased transmission of one/more plasmids at clinically and environmentally irrelevant concentrations. **Moderate risk:** increased transmission of one plasmid at clinically/environmentally concentrations. **High risk:** increased transmission of multiple plasmids at clinically/environmentally concentrations. **Very high risk:** increased transmission of multiple plasmids *in vitro* and *in vivo* at clinically/environmentally concentrations.

**Table S4.** Potential level of risk posed by environmental pollutants in disseminating antimicrobial resistance genes by horizonal gene transfer.

| **Compound (s)** | **Potential level of risk^a^** | **Evidence** | **References** |
| --- | --- | --- | --- |
| Herbicide  (glyphosate) | High | Environmentally relevant concentrations (0.1–0.6 µg/mL) increased conjugative transfer of an unnamed plasmid. | (16, 63) |
|  |  | Sublethal concentrations of glyphosate within application levels increased persistence, tolerance, and resistance of bacteria to antibiotics. | (64, 65, 66, 67) |
|  |  | Inhibited growth of 225 *Salmonella enterica* isolates at 10–80 mg/mL. | (68) |
| Disinfection by-products | High | Environmentally relevant concentrations (0.01–0.025 µg/mL) of trichloromethane and dichloroacetonitrile increased conjugative transfer of a plasmid carrying AMR genes. | (17) |
|  |  | Environmentally relevant concentrations of chlorite and iodoacetic acid had antibacterial effects and induced antibiotic resistance in *E. coli*. | (69) |
| Water treatment by-product (released nitric oxide from sodium nitroprusside) | Moderate | Concentrations of nitric oxide reported in practical wastewater treatment plants (0.031–1.04 µM) increased conjugative transfer of a plasmid carrying AMR genes between typical wastewater bacteria. | (18) |
|  |  | Nitric oxide alone can eradicate broad range of bacteria in planktonic and biofilm form. When used in tandem with antibiotics, it increases their efficacy. | (70) |
| Plastic manufacturing precursors (bisphenols) | Moderate | Environmentally relevant concentrations (0.1–100 µg/L) of bisphenol S and AF increased conjugative transfer of a plasmid carrying AMR genes. | (19) |
| Dyeing wastewater compounds | Moderate | Environmentally relevant concentrations of *o*-xylene (0.8 µg/L) and ethylbenzene (1 µg/L) increased conjugative transfer of a plasmid carrying AMR genes. | (20, 71) |
| Nanomaterials | High | Increased conjugative transfer of several plasmids carrying AMR genes between the same species of bacteria and across genera. | (24) |
|  |  | Induced persister formation in *E. coli* by promoting ROS overproduction. | (72) |
| Ionic liquids | Low | Increased conjugative transfer of plasmids carrying AMR genes from *E. coli* to a freshwater bacterial community at 1 mg/mL, which is significantly higher than environmental concentrations. Currently, not used widely in industrial applications. | (25, 26, 27, 73) |
| Multiwalled carbon nanotubes | Insufficient data | Increased conjugative transfer of two different plasmids with AMR genes at 10 µg/mL. However, very limited data is available for environmental levels of multiwalled carbon nanotubes as they are difficult to distinguish from carbon in the environment. | (28) |
| Microplastics | High | 500 microplastic particles per litre increased conjugative transfer of a plasmid carrying AMR genes, which is within the range of reported levels found in treated drinking water (338–628 particles/L). | (29, 74, 75) |
|  |  | Activated sludge from wastewater treatment plants supplemented with 1200 microplastic particles per litre enriched for antibiotic resistance genes. | (76) |
| Arsenic | Very high | Environmentally relevant concentrations of arsenic (0.5–5 µg/mL) increased conjugative transfer of several clinical AMR plasmids. | (37, 77, 78) |
|  |  | Rural Bangladeshi populations living in high arsenic-contaminated (>0.1 µg/mL) areas had significantly higher faecal carriage of multidrug resistant *E. coli* compared to populations living in low arsenic-contaminated areas (<0.02 µg/mL). | (79) |
|  |  | Environmentally relevant concentrations of arsenic (7 µg/mL) selected for a clinical extended-spectrum β-lactamase plasmid. | (80) |
| Copper | High | Environmentally relevant concentrations of copper (5 µg/L) increased conjugative transfer of plasmids carrying AMR genes. | (35, 77, 81) |
| Mercury | High | Environmentally relevant concentrations of mercury (0.1 µg/mL) increased conjugative transfer of plasmids carrying AMR genes. | (31, 82) |
|  |  | Environmentally relevant concentrations of mercury (1.9 µg/mL) induced multiple drug resistance in *E. coli*. | (83) |
|  |  | Environmentally relevant concentrations of mercury co-selected for antibiotic resistance genes in soil communities and wastewater treatment plants. | (84, 85, 86) |
| Lead | Moderate | Increased conjugative transfer of a plasmid carrying AMR genes from *E. coli* to an activated sludge community at environmentally reported concentrations (165.6 µg/mL). | (34, 87) |

^a^**Low risk:** increased transmission of one/more plasmids at clinically and environmentally irrelevant concentrations. **Moderate risk:** increased transmission of one plasmid at clinically/environmentally concentrations. **High risk:** increased transmission of multiple plasmids at clinically/environmentally concentrations. **Very high risk:** increased transmission of multiple plasmids *in vitro* and *in vivo* at clinically/environmentally concentrations.

**References**

1. Ding P, Lu J, Wang Y, Schembri MA, Guo J. Antidepressants promote the spread of antibiotic resistance via horizontally conjugative gene transfer. Environmental Microbiology. 2022;n/a(n/a).

2. Lu J, Ding P, Wang Y, Guo J. Antidepressants promote the spread of extracellular antibiotic resistance genes via transformation. ISME Communications. 2022;2(1):63.

3. Wang Y, Yu Z, Ding P, Lu J, Mao L, Ngiam L, et al. Antidepressants can induce mutation and enhance persistence toward multiple antibiotics. Proc Natl Acad Sci U S A. 2023;120(5):e2208344120.

4. Wang Y, Lu J, Engelstädter J, Zhang S, Ding P, Mao L, et al. Non-antibiotic pharmaceuticals enhance the transmission of exogenous antibiotic resistance genes through bacterial transformation. The ISME Journal. 2020;14(8):2179-96.

5. Wang Y, Lu J, Zhang S, Li J, Mao L, Yuan Z, et al. Non-antibiotic pharmaceuticals promote the transmission of multidrug resistance plasmids through intra- and intergenera conjugation. The ISME Journal. 2021;15(9):2493-508.

6. Wang Y, Yu Z, Ding P, Lu J, Klümper U, Murray AK, et al. Non-antibiotic pharmaceuticals promote conjugative plasmid transfer at a community-wide level. Microbiome. 2022;10(1):124.

7. Jia Y, Wang Z, Fang D, Yang B, Li R, Liu Y. Acetaminophen promotes horizontal transfer of plasmid-borne multiple antibiotic resistance genes. Science of The Total Environment. 2021;782:146916.

8. Cui Y, Gao J, Guo Y, Li Z, Wang Z, Zhao Y. Unraveling the impact and mechanism of antipyretic paracetamol on intergenera conjugative plasmid transfer. Environmental Research. 2022:114263.

9. Wang Y, Lu J, Mao L, Li J, Yuan Z, Bond PL, et al. Antiepileptic drug carbamazepine promotes horizontal transfer of plasmid-borne multi-antibiotic resistance genes within and across bacterial genera. The ISME Journal. 2019;13(2):509-22.

10. Yang B, Wang Z, Jia Y, Fang D, Li R, Liu Y. Paclitaxel and its derivative facilitate the transmission of plasmid-mediated antibiotic resistance genes through conjugative transfer. Science of The Total Environment. 2022;810:152245.

11. Yu Z, Wang Y, Henderson IR, Guo J. Artificial sweeteners stimulate horizontal transfer of extracellular antibiotic resistance genes through natural transformation. The ISME Journal. 2022;16(2):543-54.

12. Yu Z, Wang Y, Lu J, Bond PL, Guo J. Nonnutritive sweeteners can promote the dissemination of antibiotic resistance through conjugative gene transfer. The ISME Journal. 2021;15(7):2117-30.

13. Yu Z, Henderson IR, Guo J. Non-caloric artificial sweeteners modulate conjugative transfer of multi-drug resistance plasmid in the gut microbiota. Gut Microbes. 2023;15(1):2157698.

14. Mc Mahon MA, Blair IS, Moore JE, Mc Dowell DA. The rate of horizontal transmission of antibiotic resistance plasmids is increased in food preservation-stressed bacteria. J Appl Microbiol. 2007;103(5):1883-8.

15. Cen T, Zhang X, Xie S, Li D. Preservatives accelerate the horizontal transfer of plasmid-mediated antimicrobial resistance genes via differential mechanisms. Environment International. 2020;138:105544.

16. Zhang H, Liu J, Wang L, Zhai Z. Glyphosate escalates horizontal transfer of conjugative plasmid harboring antibiotic resistance genes. Bioengineered. 2021;12(1):63-9.

17. He K, Xue B, Yang X, Wang S, Li C, Zhang X, et al. Low-concentration of trichloromethane and dichloroacetonitrile promote the plasmid-mediated horizontal transfer of antibiotic resistance genes. Journal of Hazardous Materials. 2022;425:128030.

18. Huang H, Feng G, Wang M, Liu C, Wu Y, Dong L, et al. Nitric Oxide: A Neglected Driver for the Conjugative Transfer of Antibiotic Resistance Genes among Wastewater Microbiota. Environmental Science & Technology. 2022;56(10):6466-78.

19. Feng M, Ye C, Zhang S, Sharma VK, Manoli K, Yu X. Bisphenols promote the conjugative transfer of antibiotic resistance genes without damaging cell membrane. Environmental Chemistry Letters. 2022;20(3):1553-60.

20. Jiao YN, Chen H, Gao RX, Zhu YG, Rensing C. Organic compounds stimulate horizontal transfer of antibiotic resistance genes in mixed wastewater treatment systems. Chemosphere. 2017;184:53-61.

21. Lu J, Wang Y, Jin M, Yuan Z, Bond P, Guo J. Both silver ions and silver nanoparticles facilitate the horizontal transfer of plasmid-mediated antibiotic resistance genes. Water Res. 2020;169:115229.

22. Zhang S, Wang Y, Song H, Lu J, Yuan Z, Guo J. Copper nanoparticles and copper ions promote horizontal transfer of plasmid-mediated multi-antibiotic resistance genes across bacterial genera. Environ Int. 2019;129:478-87.

23. Qiu Z, Shen Z, Qian D, Jin M, Yang D, Wang J, et al. Effects of nano-TiO2 on antibiotic resistance transfer mediated by RP4 plasmid. Nanotoxicology. 2015;9(7):895-904.

24. Qiu Z, Yu Y, Chen Z, Jin M, Yang D, Zhao Z, et al. Nanoalumina promotes the horizontal transfer of multiresistance genes mediated by plasmids across genera. Proc Natl Acad Sci U S A. 2012;109(13):4944-9.

25. Wang Q, Mao D, Mu Q, Luo Y. Enhanced horizontal transfer of antibiotic resistance genes in freshwater microcosms induced by an ionic liquid. PLoS One. 2015;10(5):e0126784.

26. Wang X, Chen Z, Mu Q, Wu X, Zhang J, Mao D, et al. Ionic Liquid Enriches the Antibiotic Resistome, Especially Efflux Pump Genes, Before Significantly Affecting Microbial Community Structure. Environ Sci Technol. 2020;54(7):4305-15.

27. Wang Q, Lu Q, Mao D, Cui Y, Luo Y. The horizontal transfer of antibiotic resistance genes is enhanced by ionic liquid with different structure of varying alkyl chain length. Front Microbiol. 2015;6:864.

28. Weise K, Winter L, Fischer E, Kneis D, de la Cruz Barron M, Kunze S, et al. Multiwalled Carbon Nanotubes Promote Bacterial Conjugative Plasmid Transfer. Microbiol Spectr. 2022;10(2):e0041022.

29. Arias-Andres M, Klumper U, Rojas-Jimenez K, Grossart HP. Microplastic pollution increases gene exchange in aquatic ecosystems. Environ Pollut. 2018;237:253-61.

30. Suzuki S, Kimura M, Agusa T, Rahman HM. Vanadium accelerates horizontal transfer of *tet*(M) gene from marine *Photobacterium* to *Escherichia coli*. FEMS Microbiol Lett. 2012;336(1):52-6.

31. Li W, Zhang WG, Zhang MS, Lei ZF, Li PF, Ma Y, et al. Environmentally relevant concentrations of mercury facilitate the horizontal transfer of plasmid-mediated antibiotic resistance genes. Sci Total Environ. 2022;852:158272.

32. Zhang Y, Gu AZ, Cen T, Li X, He M, Li D, et al. Sub-inhibitory concentrations of heavy metals facilitate the horizontal transfer of plasmid-mediated antibiotic resistance genes in water environment. Environ Pollut. 2018;237:74-82.

33. Tang H, Liu Z, Hu B, Zhu L. Effects of iron mineral adhesion on bacterial conjugation: Interfering the transmission of antibiotic resistance genes through an interfacial process. Journal of Hazardous Materials. 2022;435:128889.

34. Lin H, Jiang L, Li B, Dong Y, He Y, Qiu Y. Screening and evaluation of heavy metals facilitating antibiotic resistance gene transfer in a sludge bacterial community. Sci Total Environ. 2019;695:133862.

35. Wang Q, Liu L, Hou Z, Wang L, Ma D, Yang G, et al. Heavy metal copper accelerates the conjugative transfer of antibiotic resistance genes in freshwater microcosms. Sci Total Environ. 2020;717:137055.

36. Pu Q, Fan XT, Li H, An XL, Lassen SB, Su JQ. Cadmium enhances conjugative plasmid transfer to a fresh water microbial community. Environ Pollut. 2021;268(Pt B):115903.

37. Kothari A, Kumar P, Gaurav A, Kaushal K, Pandey A, Yadav SRM, et al. Association of antibiotics and heavy metal arsenic to horizontal gene transfer from multidrug-resistant clinical strains to antibiotic-sensitive environmental strains. Journal of Hazardous Materials. 2023;443:130260.

38. McGovern AS, Hamlin AS, Winter G. A review of the antimicrobial side of antidepressants and its putative implications on the gut microbiome. Aust N Z J Psychiatry. 2019;53(12):1151-66.

39. Ou J, Elizalde P, Guo HB, Qin H, Tobe BTD, Choy JS. TCA and SSRI Antidepressants Exert Selection Pressure for Efflux-Dependent Antibiotic Resistance Mechanisms in *Escherichia coli*. mBio. 2022;13(6):e0219122.

40. Munoz-Bellido JL, Munoz-Criado S, Garcı̀a-Rodrı̀guez JA. Antimicrobial activity of psychotropic drugs: Selective serotonin reuptake inhibitors. International Journal of Antimicrobial Agents. 2000;14(3):177-80.

41. Rukavishnikov G, Leonova L, Kasyanov E, Leonov V, Neznanov N, Mazo G. Antimicrobial activity of antidepressants on normal gut microbiota: Results of the *in vitro* study. Front Behav Neurosci. 2023;17:1132127.

42. Davies NM, Anderson KE. Clinical pharmacokinetics of diclofenac. Therapeutic insights and pitfalls. Clin Pharmacokinet. 1997;33(3):184-213.

43. Li X, Xue X, Jia J, Zou X, Guan Y, Zhu L, et al. Nonsteroidal anti-inflammatory drug diclofenac accelerates the emergence of antibiotic resistance via mutagenesis. Environ Pollut. 2023;326:121457.

44. Dutta NK, Annadurai S, Mazumdar K, Dastidar SG, Kristiansen JE, Molnar J, et al. Potential management of resistant microbial infections with a novel non-antibiotic: the anti-inflammatory drug diclofenac sodium. International Journal of Antimicrobial Agents. 2007;30(3):242-9.

45. Salem-Milani A, Balaei-Gajan E, Rahimi S, Moosavi Z, Abdollahi A, Zakeri-Milani P, et al. Antibacterial Effect of Diclofenac Sodium on *Enterococcus faecalis*. J Dent (Tehran). 2013;10(1):16-22.

46. Davies NM, Anderson KE. Clinical Pharmacokinetics of Naproxen. Clinical Pharmacokinetics. 1997;32(4):268-93.

47. Janssen GM, Venema JF. Ibuprofen: plasma concentrations in man. J Int Med Res. 1985;13(1):68-73.

48. Zur J, Pinski A, Marchlewicz A, Hupert-Kocurek K, Wojcieszynska D, Guzik U. Organic micropollutants paracetamol and ibuprofen-toxicity, biodegradation, and genetic background of their utilization by bacteria. Environ Sci Pollut Res Int. 2018;25(22):21498-524.

49. Verma T, Bhaskarla C, Sadhir I, Sreedharan S, Nandi D. Non-steroidal anti-inflammatory drugs, acetaminophen and ibuprofen, induce phenotypic antibiotic resistance in E*scherichia coli*: roles of *marA* and *acrB*. FEMS Microbiol Lett. 2018;365(22).

50. Shah PN, Marshall-Batty KR, Smolen JA, Tagaev JA, Chen Q, Rodesney CA, et al. Antimicrobial Activity of Ibuprofen against Cystic Fibrosis-Associated Gram-Negative Pathogens. Antimicrob Agents Chemother. 2018;62(3).

51. Brett CN, Barnett SG, Pearson J. Postoperative plasma paracetamol levels following oral or intravenous paracetamol administration: a double-blind randomised controlled trial. Anaesth Intensive Care. 2012;40(1):166-71.

52. Knauf H, Kolle EU, Mutschler E. Gemfibrozil absorption and elimination in kidney and liver disease. Klin Wochenschr. 1990;68(13):692-8.

53. Korkmaz NE, Savun-Hekimoğlu B, Aksu A, Burak S, Caglar NB. Occurrence, sources and environmental risk assessment of pharmaceuticals in the Sea of Marmara, Turkey. Science of The Total Environment. 2022;819:152996.

54. Fang Y, Karnjanapiboonwong A, Chase DA, Wang J, Morse AN, Anderson TA. Occurrence, fate, and persistence of gemfibrozil in water and soil. Environ Toxicol Chem. 2012;31(3):550-5.

55. Sumpter JP, Runnalls TJ, Donnachie RL, Owen SF. A comprehensive aquatic risk assessment of the beta-blocker propranolol, based on the results of over 600 research papers. Science of The Total Environment. 2021;793:148617.

56. Nolen WA, Jansen GS, Broekman M. Measuring plasma levels of carbamazepine. A pharmacokinetic study in patients with affective disorders. Pharmacopsychiatry. 1988;21(5):252-4.

57. Batucan NSP, Tremblay LA, Northcott GL, Matthaei CD. Medicating the environment? A critical review on the risks of carbamazepine, diclofenac and ibuprofen to aquatic organisms. Environmental Advances. 2022;7:100164.

58. Wang Y-F, Qiao M, Zhu D, Zhu Y-G. Antibiotic Resistance in the Collembolan Gut Microbiome Accelerated by the Nonantibiotic Drug Carbamazepine. Environmental Science & Technology. 2020;54(17):10754-62.

59. Stage TB, Bergmann TK, Kroetz DL. Clinical Pharmacokinetics of Paclitaxel Monotherapy: An Updated Literature Review. Clin Pharmacokinet. 2018;57(1):7-19.

60. Wormington AM, De Maria M, Kurita HG, Bisesi JH, Jr., Denslow ND, Martyniuk CJ. Antineoplastic Agents: Environmental Prevalence and Adverse Outcomes in Aquatic Organisms. Environ Toxicol Chem. 2020;39(5):967-85.

61. FDA. Additional Information about High-Intensity Sweeteners Permitted for Use in Food in the United States: U.S. Food & Drug Administration; 2018 [Available from: <https://www.fda.gov/food/food-additives-petitions/additional-information-about-high-intensity-sweeteners-permitted-use-food-united-states>.

62. de Dios R, Proctor CR, Maslova E, Dzalbe S, Rudolph CJ, McCarthy RR. Artificial sweeteners inhibit multidrug-resistant pathogen growth and potentiate antibiotic activity. EMBO Mol Med. 2022:e16397.

63. Peruzzo PJ, Porta AA, Ronco AE. Levels of glyphosate in surface waters, sediments and soils associated with direct sowing soybean cultivation in north pampasic region of Argentina. Environ Pollut. 2008;156(1):61-6.

64. Ospino K, Spira B. Glyphosate affects persistence and tolerance but not antibiotic resistance. BMC Microbiology. 2023;23(1):61.

65. Kurenbach B, Marjoshi D, Amábile-Cuevas CF, Ferguson GC, Godsoe W, Gibson P, et al. Sublethal Exposure to Commercial Formulations of the Herbicides Dicamba, 2,4-Dichlorophenoxyacetic Acid, and Glyphosate Cause Changes in Antibiotic Susceptibility in *Escherichia coli* and *Salmonella enterica* serovar Typhimurium. mBio. 2015;6(2):e00009-15.

66. Xing Y, Wu S, Men Y. Exposure to Environmental Levels of Pesticides Stimulates and Diversifies Evolution in *Escherichia coli* toward Higher Antibiotic Resistance. Environmental Science & Technology. 2020;54(14):8770-8.

67. Liao H, Li X, Yang Q, Bai Y, Cui P, Wen C, et al. Herbicide Selection Promotes Antibiotic Resistance in Soil Microbiomes. Molecular Biology and Evolution. 2021;38(6):2337-50.

68. Poppe J, Bote K, Merle R, Makarova O, Roesler U. Minimum Inhibitory Concentration of Glyphosate and a Glyphosate-Containing Herbicide in *Salmonella enterica* Isolates Originating from Different Time Periods, Hosts, and Serovars. Eur J Microbiol Immunol (Bp). 2019;9(2):35-41.

69. Li D, Zeng S, He M, Gu AZ. Water Disinfection Byproducts Induce Antibiotic Resistance-Role of Environmental Pollutants in Resistance Phenomena. Environ Sci Technol. 2016;50(6):3193-201.

70. Rouillard KR, Novak OP, Pistiolis AM, Yang L, Ahonen MJR, McDonald RA, et al. Exogenous Nitric Oxide Improves Antibiotic Susceptibility in Resistant Bacteria. ACS Infectious Diseases. 2021;7(1):23-33.

71. Duan W, Meng F, Wang F, Liu Q. Environmental behavior and eco-toxicity of xylene in aquatic environments: A review. Ecotoxicol Environ Saf. 2017;145:324-32.

72. Wang S, Zhao C, Xue B, Li C, Zhang X, Yang X, et al. Nanoalumina triggers the antibiotic persistence of *Escherichia coli* through quorum sensing regulators *lrsF* and *qseB*. Journal of Hazardous Materials. 2022;436:129198.

73. Pham TP, Cho CW, Yun YS. Environmental fate and toxicity of ionic liquids: a review. Water Res. 2010;44(2):352-72.

74. Zhang G, Chen J, Li W. Conjugative antibiotic-resistant plasmids promote bacterial colonization of microplastics in water environments. J Hazard Mater. 2022;430:128443.

75. Pivokonsky M, Cermakova L, Novotna K, Peer P, Cajthaml T, Janda V. Occurrence of microplastics in raw and treated drinking water. Science of The Total Environment. 2018;643:1644-51.

76. Pham DN, Clark L, Li M. Microplastics as hubs enriching antibiotic-resistant bacteria and pathogens in municipal activated sludge. Journal of Hazardous Materials Letters. 2021;2:100014.

77. Palm M, Fransson A, Hulten J, Bucaro Stenman K, Allouche A, Chiang OE, et al. The Effect of Heavy Metals on Conjugation Efficiency of an F-Plasmid in *Escherichia coli*. Antibiotics (Basel). 2022;11(8).

78. Smedley PL, Kinniburgh DG. A review of the source, behaviour and distribution of arsenic in natural waters. Applied Geochemistry. 2002;17(5):517-68.

79. Amin MB, Talukdar PK, Asaduzzaman M, Roy S, Flatgard BM, Islam MR, et al. Effects of chronic exposure to arsenic on the fecal carriage of antibiotic-resistant *Escherichia coli* among people in rural Bangladesh. Plos Pathog. 2022;18(12):e1010952.

80. Gullberg E, Albrecht LM, Karlsson C, Sandegren L, Andersson DI. Selection of a multidrug resistance plasmid by sublethal levels of antibiotics and heavy metals. mBio. 2014;5(5):e01918-14.

81. ECCC. Federal environmental quality guidelines - Copper. Canada: Environment and Climate Change Canada; 2021.

82. Obasi PN, Akudinobi BB. Potential health risk and levels of heavy metals in water resources of lead–zinc mining communities of Abakaliki, southeast Nigeria. Applied Water Science. 2020;10(7):184.

83. Fuentes AM, Amábile-Cuevas CF. Mercury induces multiple antibiotic resistance in *Escherichia coli* through activation of SoxR, a redox-sensing regulatory protein. FEMS Microbiology Letters. 1997;154(2):385-8.

84. Zhao Y, Hu H-W, Su J-Q, Hao X, Guo H, Liu Y-R, et al. Influence of Legacy Mercury on Antibiotic Resistomes: Evidence from Agricultural Soils with Different Cropping Systems. Environmental Science & Technology. 2021;55(20):13913-22.

85. Mahbub KR, King WL, Siboni N, Nguyen VK, Rahman MM, Megharaj M, et al. Long-lasting effect of mercury contamination on the soil microbiota and its co-selection of antibiotic resistance. Environmental Pollution. 2020;265:115057.

86. Czekalski N, Gascón Díez E, Bürgmann H. Wastewater as a point source of antibiotic-resistance genes in the sediment of a freshwater lake. The ISME Journal. 2014;8(7):1381-90.

87. Yang QW, Shu WS, Qiu JW, Wang HB, Lan CY. Lead in paddy soils and rice plants and its potential health risk around Lechang Lead/Zinc Mine, Guangdong, China. Environment International. 2004;30(7):883-9.
